# ﻿The era of cybertaxonomy: X-ray microtomography reveals cryptic diversity and concealed cuticular sculpture in *Aphanerostethus* Voss, 1957 (Coleoptera, Curculionidae)

**DOI:** 10.3897/zookeys.1217.126626

**Published:** 2024-10-29

**Authors:** Jake H. Lewis, Hiroaki Kojima, Miyuki Suenaga, Dimitrios Petsopoulos, Yusuke Fujisawa, Xuan Lam Truong, Dan L. Warren

**Affiliations:** 1 Environmental Science and Informatics Section, Okinawa Institute of Science and Technology Graduate University, 1919-1 Tancha, Onna-son, Kunigami-gun, Okinawa, 904-0495 Japan; 2 Department of Natural History, New Brunswick Museum, 277 Douglas Avenue, Saint John, New Brunswick, E2K 1E5, Canada; 3 Laboratory of Entomology, Tokyo University of Agriculture, 1737 Funako, Atsugi, Kanagawa, 243-0034, Japan; 4 Biodiversity and Biocomplexity Unit, Okinawa Institute of Science and Technology Graduate University, 1919-1 Tancha, Onna-son, Kunigami-gun, Okinawa, 904-0495, Japan; 5 Shonan Fujisawa Junior and Senior High School, Keio University, 5466 Endo, Fujisawa, Kanagawa Prefecture, 252-0816 Japan; 6 Institute of Ecology & Biological Resources, Vietnamese Academy of Science & Technology, 18 Hoang Quoc Viet Road, Cau Giay, Ha Noi, Vietnam; 7 Gulbali Institute for Applied Ecology, Charles Sturt University, Albury, NSW, 2640, Australia

**Keywords:** Biodiversity, DNA barcoding, integrative taxonomy, μCT, micro-ct, sexual selection, species discovery, weevil

## Abstract

Weevils represent one of the most speciose and economically important animal clades, but remain poorly studied across much of the Oriental Region. Here, an integrative revision of the Oriental, flightless genus *Aphanerostethus* Voss, 1957 (Curculionidae: Molytinae) based on X-ray microtomography, multi-gene DNA barcoding (CO1, Cytb, 16S), and traditional morphological techniques (light microscopy, dissections) is presented. Twelve new species, namely, *A.armatus* Lewis & Kojima, **sp. nov.**, *A.bifidus* Kojima & Lewis, **sp. nov.**, *A.darlingi* Lewis, **sp. nov.**, *A.decoratus* Lewis & Kojima, **sp. nov.**, *A.falcatus* Kojima, Lewis & Fujisawa, **sp. nov.**, *A.incurvatus* Kojima & Lewis, **sp. nov.**, *A.japonicus* Lewis & Kojima, **sp. nov.**, *A.magnus* Lewis & Kojima, **sp. nov.**, *A.morimotoi* Kojima & Lewis, **sp. nov.**, *A.nudus* Lewis & Kojima, **sp. nov.**, *A.spinosus* Lewis & Kojima, **sp. nov.**, and *A.taiwanus* Lewis, Fujisawa & Kojima, **sp. nov.** are described from Japan, Taiwan, Vietnam, and Malaysia. A neotype is designated for *A.vannideki* Voss, 1957. The hitherto monotypic genus *Darumazo* Morimoto & Miyakawa, 1985, **syn. nov.** is synonymized under *Aphanerostethus* based on new morphological data and *Aphanerostethusdistinctus* (Morimoto & Miyakawa, 1985), **comb. nov.** is transferred accordingly. X-ray microtomography is successfully used to explore for stable interspecific differences in cuticular, internal and micro morphology. Remarkable species-specific sexual dimorphism in the metatibial uncus is described in seven of the newly described *Aphanerostethus* species and the evolution of this character is discussed.

## ﻿Introduction

Weevils (Coleoptera: Curculionoidea) represent one of the most diverse animal groups (ca 62,000 species) and include many economically important pest species (Oberprieler et al. 2014). The great diversity of weevils has been attributed to their co-evolution with Angiosperms (McKenna et al. 2009; Winter et al. 2017), and they have not only evolved to feed on living plants, but also dead plants, fungi, and even mammal dung (Jordal and Cognato 2012; Oberprieler et al. 2014; Escalona et al. 2023). Although many generalists with wide host-plant ranges exist, highly specialized species only feed on one plant species or genus (Anderson 1993; Young et al. 2008). Specialist weevil species are at risk when their host plant is threatened as they lack the ability to switch to other food sources. This has led to presumed extinctions in weevils, such as the Greater Chestnut Weevil *Curculiocaryatrypes* (Boheman, 1843) which fed only on American Chestnut (*Castaneadentata* (Marsh.) Borkh.) before the introduced fungal pathogen *Cryphonectriaparasitica* (Murrill) M.E.Barr decimated chestnut populations across the United States (Charles and McKenna 2023). Weevil conservation relies most heavily on a solid understanding of host plant preferences, which often differ significantly between species even belonging to the same genus (e.g., Wibmer 1981; Yoshihara 2016; Lewis and Anderson 2022). For this reason, accurate species delineation of weevils (and their host plants) is essential to the discovery of interspecific differences in host plant, and is one of the most fundamental components of conservation biology.

As a tropical mountainous archipelago, Southeast Asia is remarkable for its weevil diversity and high rates of endemism (Setliff 2007; Tanzler et al. 2014; Sprick and Floren 2018). The complex geologic history of southeast Asia has positively influenced weevil speciation rates (Tänzler et al. 2016; Tseng et al. 2017; Letsch et al. 2023), and the loss of flight (common in weevils) undoubtedly contributed further to this radiation (Ikeda et al. 2012; Tänzler et al. 2014). Furthermore, some evidence exists suggesting that tropical *Curculio* are more specialized in host-plant usage than species in temperate zones (Peguero et al. 2017), and this has been suggested as another explanation of the great diversity of phytophagous insects in tropical zones (Forister et al. 2014). Unfortunately for conservation and taxonomy, southeast Asia is also experiencing extreme deforestation (Estoque et al. 2019), and satellite data suggests that one of its most biodiverse regions, Borneo and Sumatra, experienced especially high rates of forest loss from 2000 to 2010 (Achard et al. 2014). Weevil species richness (and beetle species richness in general) is negatively correlated with logging activity and anthropogenic disturbance (Su et al. 2011; Hagge et al. 2019; Sharp et al. 2019), which is certainly at least partially attributable to decreases in plant diversity. Natural history collections and especially those with a focus on tropical fauna have never been more valuable as windows into the past. Important to the preservation of natural history collections is the development and use of non-destructive techniques such as X-ray microtomography and BOMB DNA extraction (Oberacker et al. 2019), which allow for analyses of specimens without sacrificing precious type, historical, or rare material. Such non-destructive methods will be increasingly valuable going forward, as specimen acquisition is impeded by species loss due to habitat destruction and a greater prevalence of opaque biodiversity protection laws.

The oriental, flightless weevil genus *Aphanerostethus* Voss, 1957 was described based on a single species, *A.vannideki*, from West Java, Indonesia, and has been distinguished from similar apterous genera by their daruma doll (hence the name *Darumazo* Morimoto & Miyakawa, 1985, syn. nov.) to bulb-like shape, the six-articled funicle, a canaliculate prosternum, erect scales between eyes (“eye-lashed” appearance), and large eyes (Voss 1957; Morimoto and Miyakawa 1985; Morimoto 2011). The genus is ecologically poorly studied; however, one species, *A.distinctus* (Morimoto & Miyakawa, 1985), has been reared from the galls of *Asteralobiasasakii* (Monzen, 1937) (Diptera: Cecidomyiidae) on IlexcrenataThunb.var.hachijoensis Nakai (Aquifoliaceae), and *Ilexintegra* Thunb. (Fujii et al. 2012; Kojima 2013), suggesting that (at least) *A.distinctus* may have a highly specialized cecidophagous lifestyle. Although cecidophagy has been reported in a number of lineages (e.g., Yamazaki and Sugiura 2001; Wilson 2012; Prena 2021), it is relatively uncommon in weevils. Here, *Aphanerostethus* Voss, 1957 (= *Darumazo* Morimoto & Miyakawa, 1985, syn. nov.) (Coleoptera: Curculionidae: Molytinae) is revised using a combination of X-ray microtomography, multi-gene DNA barcoding (CO1, Cytb, 16S), and traditional morphological techniques (light microscopy, dissections). Twelve new species of *Aphanerostethus* are described based on specimens collected in Japan, Taiwan, Vietnam, and Malaysia, and seven of these exhibit stunning sexual dimorphism and species-specific variation in the metatibial unci. X-ray microtomography (hereafter X-ray μCT) has become a widely used tool in entomology and has broad applications including enhancing analysis of insects enclosed in amber (Kundrata et al. 2020; Kypke and Solodovnikov 2020), examination of internal morphology (Alba-Alejandre et al. 2019; Aibekova et al. 2022), and for taxonomic character discovery (Garcia et al. 2017; Lewis 2023). The great advantage of X-ray μCT is that it allows for a complete three-dimensional viewing of minute internal and external structures, including those obscured by musculature, dirt and debris, or scales, and that 3D models can be uploaded to online databases for viewing by anyone with access to a computer. X-ray μCT is ideal when working with primary type material, precious fossils, or rare species, as it does not require dissection or intrusive manipulation. As is common in many weevil groups, most *Aphanerostethus* species are covered in a dense mat of appressed scales which obscure the underlying cuticle. By removing scales during the segmentation process, Lewis (2023) found apparent interspecific differences in cuticular sculpturing in the weevil genus *Karekizo* Morimoto, 1962, but was severely limited in sample size (one specimen of each species). As such, it was impossible to ascertain whether these hidden cuticular characters were intraspecifically stable and thereby useful for taxonomy and phylogenetics. We use the same method of Lewis (2023) to search for interspecific differences in pronotal and elytral surface structure as well as internal morphology (hindwing reduction), and investigate the stability of these characters by expanding the sample size to five specimens per species to account for any intraspecific variation. We also use X-ray μCT to examine and visualize the minute metatibial unci of *Aphanerostethus*, which are less than 50 μm in length and frequently obscured by scales and hairs.

## ﻿Materials and methods

### ﻿Specimen acquisition and general methodology

Specimens were examined from the following collections:

**CMNC**Canadian Museum of Nature, Gatineau, Canada

**ELKU**Entomology Lab of Kyushu University, Fukuoka, Japan

**FFPRI**Forestry and Forest Products Research Institute, Tsukuba, Japan

**HUM**Hokkaido University Museum, Sapporo, Japan

**KUM**Kyushu University Museum, Fukuoka, Japan

**NMNST**National Museum of Natural Science, Taichung, Taiwan

**OIST** Okinawa Institute of Science and Technology, Tancha, Japan

**PCHY** Private Collection of H. Yoshitake, Tsukuba, Japan

**RMNH**"Naturalis Biodiversity Centre, Leiden, Netherlands

**RUMC** Ryukyu University Museum Collection, Nishihara, Japan

**SFDK**Sarawak Forestry Department, Kuching, Malaysia

**TARI**Taiwan Agricultural Research Institute, Taichung, Taiwan

**TUA**Tokyo University of Agriculture, Atsugi, Japan

**ZMH**Zoological Museum Hamburg, Hamburg, Germany

All examined specimens without institutional Unique Specimen Identifier (USI) labels were assigned labels that read in the form: JHLHY_DAR_###. Specimens were dissected following standard procedures and genitalia were cleared in a solution of KOH and water. Genitalia were photographed with a Nikon DS-Fi3 camera through a Nikon SMZ18 stereomicroscope using NIS-Elements D (v. 5.41.00) (Nikon Corporation, Yokohama, Japan), and were subsequently placed in a small tube of glycerin which was pinned with the dissected specimen. All other images were taken under a Leica M205 C microscope with a Leica DMC 5400 camera and stacked using Leica Application Suite (Leica Microsystems, Wetzlar, Germany). Figures were arranged in Adobe Photoshop (v. 24.3.0).

### ﻿X-ray microtomography

X-ray μCT scanning was performed using a ZEISS Xradia 510 Versa with ZEISS Scout and Scan Control System software (v. 14.0.14829). For scans of the metatibia, a hind leg was removed and glued to paper point and fixed to a secure mount. To compare interspecific differences in cuticle and hindwing morphology (full body scans), five specimens (when available) varying in size and collection locality were chosen in an effort to encapsulate any potential intraspecific variation. All characters presented here are those which were determined to be stable within the five-specimen sample. Obscuring scales were virtually removed from specimens by varying the threshold during segmentation and also by viewing cross-sections of the body using the *scissors* function in 3D Slicer (v. 5.0.3). All interspecific differences were confirmed by examining worn and dissected specimens. Specimens were rotated 360 degrees throughout the scan run and with 2001 projections. Reconstructions were performed using Zeiss Scout-and-Scan Control System Reconstructor (v. 14.0.14829) and saved in DICOM format. The DICOM files were loaded into 3D Slicer and 3D models were generated in the *Segment Editor* module. The 3D weevil models were cleaned to remove “noise particles” (i.e., remaining bits of scales and background noise) using the *islands* function (*Segment Editor* module) and any leftover particles were removed using the *scissors* function (*Segment Editor* module). Model shading (Cook-Torrance.gdp) was enhanced in MeshLab (v. 2022.02). Individual full body scan and metatibia scan settings for the species are found in Table 1.

**Table 1.** X-ray μCT scan settings for species of *Aphanerostethus*. An asterisk (*) beside the species name indicates that the scan was taken of the metatibia, as opposed to the full body.

Species	USI	Magnification (×)	Expo-sure (s)	Source distance (mm)	Detector distance (mm)	Volt-age (kV)	Power (W)
*Aphanerostethusarmatus*	JHLHY_DAR_078	4	0.7	13.12	10.33	50	4
A.bifidus*	JHLHY_DAR_102	20	7	11.53	8	50	4
*A.bifidus*	JHLHY_DAR_092	4	0.8	14.02	8.52	50	4
*A.bifidus*	JHLHY_DAR_101	4	0.7	11.60	6.88	50	4
*A.bifidus*	JHLHY_DAR_103	4	0.7	12.61	6.88	50	4
*A.darlingi*	JHLHY_DAR_125	4	0.7	13.04	8.01	50	4
*A.darlingi*	JHLHY_DAR_126	4	0.7	12.53	8.01	50	4
*A.decoratus*	JHLHY_DAR_079	4	1	16.02	13.40	60	4
A.distinctus*	OKENT0087658	20	5	11.07	8.04	50	4
*A.distinctus*	JHLHY_DAR_011	4	0.9	13.52	15.52	50	4
*A.distinctus*	JHLHY_DAR_062	4	0.6	11.64	7.55	60	4
*A.distinctus*	JHLHY_DAR_071	4	0.6	12.19	8.16	60	4
*A.distinctus*	OKENT0089414	4	0.7	11.53	7.57	50	4
*A.distinctus*	JHLHY_DAR_146	4	0.7	10.01	7.72	50	4
A.falcatus*	JHLHY_DAR_099	20	5	11.04	7.52	60	4
*A.falcatus*	JHLHY_DAR_091	4	0.7	12.03	8.52	50	4
*A.falcatus*	JHLHY_DAR_167	4	0.7	10.53	8.72	50	4
*A.falcatus*	JHLHY_DAR_172	4	0.7	10.53	8.22	50	4
*A.falcatus*	JHLHY_DAR_170	4	0.7	10.53	7.73	50	4
*A.falcatus*	JHLHY_DAR_094	4	0.7	10.53	8.23	50	4
A.incurvatus*	JHLHY_DAR_100	20	7	12.02	8.01	50	4
*A.incurvatus*	JHLHY_DAR_100	4	0.7	12.03	9.02	50	4
*A.incurvatus*	JHLHY_DAR_095	4	0.7	10.52	7.72	50	4
*A.incurvatus*	JHLHY_DAR_104	4	0.7	12.16	7.39	50	4
*A.japonicus*	JHLHY_DAR_051	4	1.3	16.58	15.97	60	4
*A.japonicus*	JHLHY_DAR_041	4	0.6	11.46	7.53	60	4
*A.japonicus*	JHLHY_DAR_052	4	0.65	12.08	7.54	60	4
*A.japonicus*	OKENT0055168	4	0.6	11.54	7.57	60	4
*A.japonicus*	OKENT0055232	4	0.65	11.13	7.39	50	4
A.magnus*	JHLHY_DAR_075	20	5	12.05	8.02	50	4
*A.magnus*	JHLHY_DAR_029	4	1.3	18.53	15.52	50	4
*A.magnus*	JHLHY_DAR_035	4	1	17.54	7.02	60	4
*A.magnus*	JHLHY_DAR_027	4	1	16.54	7.02	60	4
*A.magnus*	JHLHY_DAR_032	4	1	17.45	7.52	60	4
*A.magnus*	JHLHY_DAR_108	4	0.7	10.59	7.37	50	4
A.morimotoi*	JHLHY_DAR_113	20	7	11.03	8.51	50	4
*A.morimotoi*	JHLHY_DAR_093	4	0.7	11.52	9.03	50	4
*A.morimotoi*	JHLHY_DAR_188	4	0.7	11.03	7	50	4
*A.morimotoi*	JHLHY_DAR_189	4	0.7	11.04	7.51	50	4
*A.morimotoi*	JHLHY_DAR_114	4	0.7	12.13	8.14	60	4
*A.morimotoi*	JHLHY_DAR_115	4	0.7	11.56	7.51	50	4
A.nudus*	JHLHY_DAR_012	20	5	16.05	7.53	50	4
*A.nudus*	JHLHY_DAR_014	4	1.5	14.58	19.97	60	4
*A.nudus*	JHLHY_DAR_074	4	0.6	11.55	7.57	60	4
*A.nudus*	JHLHY_DAR_012	4	0.6	11.57	7.57	60	4
*A.nudus*	JHLHY_DAR_015	4	0.6	12.08	7.57	60	4
*A.nudus*	JHLHY_DAR_013	4	0.6	11.59	7.57	60	4
A.spinosus*	JHLHY_DAR_077	20	6	10.52	7.51	50	4
*A.taiwanus*	JHLHY_DAR_018	4	0.7	12.01	7.51	60	4
*A.taiwanus*	JHLHY_DAR_021	4	0.6	11.53	7.51	60	4
*A.taiwanus*	JHLHY_DAR_070	4	0.6	11.54	7.52	60	4
*A.taiwanus*	JHLHY_DAR_084	4	0.6	11.66	7.65	60	4
*A.taiwanus*	JHLHY_DAR_086	4	0.6	12.14	7.65	60	4
*A.vannideki*	JHLHY_DAR_081	4	0.7	13.67	7.65	60	4
*A.vannideki*	JHLHY_DAR_082	4	0.7	11.01	7.41	50	4
*A.vannideki*	ZMH 841853	4	0.7	12.1	8.04	60	4
*A.vannideki*	ZMA.INS.5117698	4	0.7	11.61	8.04	60	4
*A.vannideki*	ZMN.INS.5117696	4	0.7	12.62	8.04	60	4

### ﻿DNA barcoding

To complement morphology-based taxonomic hypotheses and to help with associating females with males (not trivial in the Vietnamese species), a maximum likelihood (hereafter ML) tree including eight of the 14 *Aphanerostethus* species was constructed. The Molytine weevils *Deretiosusalbicaudatus* Morimoto, 1988 (Sophrorhinini Lacordaire, 1865), *Colobodesornatoideus* Morimoto, 1988 (Sophrorhinini Lacordaire, 1865), *Protacallodesryukyuensis* Morimoto, 2011 (Ithyporini Lacordaire, 1865), *Protacallodes* sp. 1, *Ectatorhinusadamsii* Pascoe, 1871 (Ithyporini Lacordaire, 1865), and Tylodina (tribe) sp. 1 (Cryptorhynchini Schoenherr, 1825) were used as outgroup taxa (see Alonso-Zarazaga et al. 2023 for tribal placement). DNA was extracted from the whole specimen non-destructively using the methods of Oberacker et al. (2019). When used exclusively, cytochrome c oxidase subunit I (CO1) can fail to delineate closely related weevil species (Lewis and Anderson 2023; Schutte et al. 2023); as such, we sequenced ~300 bp long fragments from three mitochondrial genes, namely, 16S rRNA, CO1, and cytochrome b (Cytb). Primers and thermal profiles used are presented in Suppl. material 1. General library preparation methodology is outlined in Kennedy et al. (2023; see Section 2.3). Pooled products were sequenced with Illumina MiSeq at OIST using 600-cycle v3 kits. Sample demultiplexing within individual libraries was conducted using Cutadapt (v. 1.18; Martin 2011). Processing of DNA sequence data (de-novo assembly, trimming) was performed in Geneious Prime (v. 11.0.14.1; Dotmatics, Boston, Massachusetts, United States of America). After de-novo assembly all sequence data were run through BLAST to ensure that non-beetle DNA had not been amplified. As a final check that non-target sequence data was not erroneously incorporated into the analyses, a separate neighbor-joining tree was built for each gene to confirm that members of the same species (determined by morphology earlier in the study) clustered together as expected. DNA extraction sample codes read in the form EGP#######. All DNA sequence data were uploaded to GenBank: CO1, accession numbers PP110442–PP110480; Cytb, accession numbers PP115961–PP115995; 16S, accession numbers PP109310–PP109347. DNA sequence data was aligned using MUSCLE (v. 3.8.425 with default settings; Edgar 2004) using default settings for CO1 and Cytb, and quality-controlled by examining translated amino-acid alignments. A structural alignment algorithm (Q-INS-i with default settings; Rozewicki et al. 2019) was used to align 16S sequence data. The CO1, Cytb, and 16S fragments were concatenated into an 872 base pair long alignment which was used in the phylogenetic analysis. Except for EGP0160H08 (16S / CO1 only), EGP0160C07 (16S / CO1 only), EGP0160E02 (16S / CO1 only), and EGP0160F03 (CO1 only), all specimens had full sequence data. Partition model selection was performed in IQ-TREE (v. 1.6.12; Nguyen et al. 2015) using ModelFinder (command: -m TESTMERGE; Kalyaanamoorthy et al. 2017; Chernomor et al. 2016) to allow for separate models to be applied to the three marker fragments (CO1 / Cytb: GTR+F+I+G4; 16S: GTR+F+G4) separately. The ML analysis was performed in IQ-TREE with a heuristic search of 100,000 initial trees and standard nonparametric bootstrap (hereafter BS) values were calculated from 1000 replicates. To supplement the ML analysis, a Bayesian Inference (hereafter BI) tree was also constructed in Mr. Bayes (v. 3.2.7a; Ronquist et al. 2012) using the same partition model with the following settings (ngen: 2000000, samplefreq: 1000, nruns: 2, nchains: 4, burninfrac: 0.25). Tracer (v. 1.7.2; Rambaut et al. 2018) was used to confirm convergence of the BI analysis (ESS > 800 for all parameter values; ASDFS < 0.005). The tree presented in this paper was visualized in iTOL (v. 6.8.1; Letunic and Bork 2021) and is the ML tree with bootstrap and posterior probability (hereafter PP) values displayed at the nodes.

## ﻿Results

### ﻿General remarks

**Figure 1.**
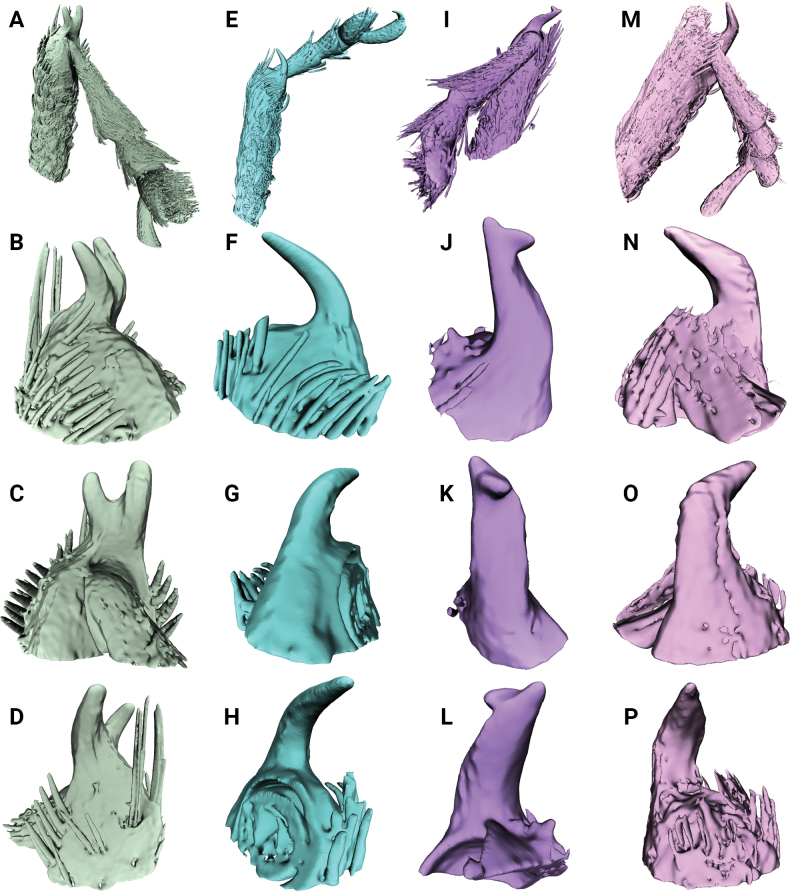
Metatibial unci in *Aphanerostethus* (males) **A–D***A.bifidus* sp. nov. (JHLHY_DAR_102) **E–H***A.distinctus* (Morimoto & Miyakawa, 1985) (OKENT0087658) **I–L***A.falcatus* sp. nov. (JHLHY_DAR_099) **M–P***A.incurvatus* sp. nov. (JHLHY_DAR_100).

**Figure 2.**
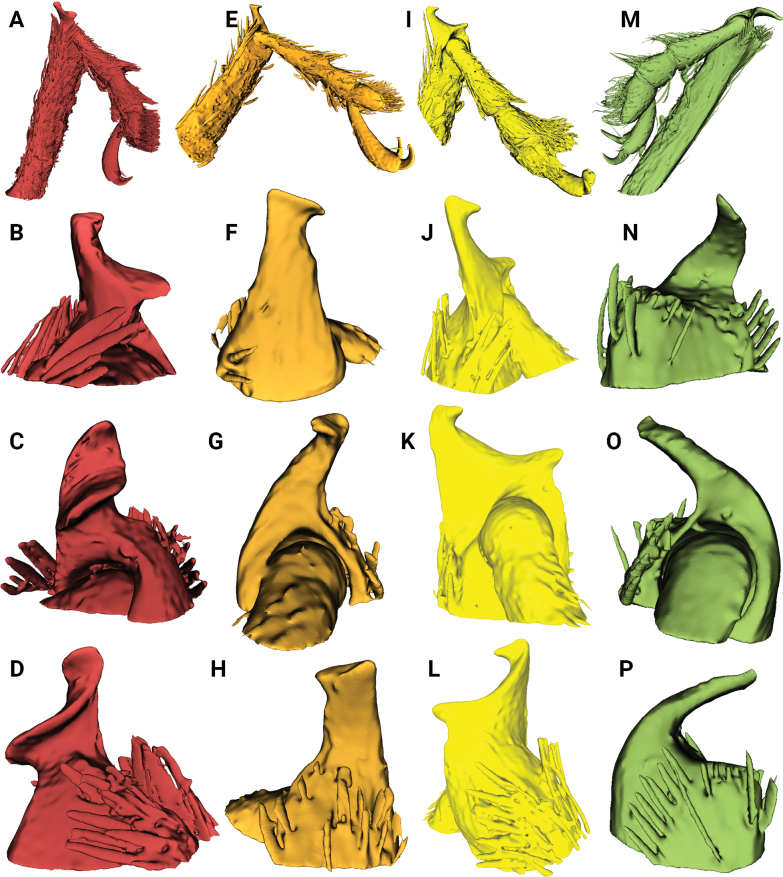
Metatibial unci in *Aphanerostethus* (males) **A–D***A.magnus* sp. nov. (JHLHY_DAR_075) **E–H***A.morimotoi* sp. nov. (JHLHY_DAR_113) **I–L***A.spinosus* sp. nov. (JHLHY_DAR_077) **M–P***A.nudus* sp. nov. (JHLHY_DAR_012).

**Figure 3.**
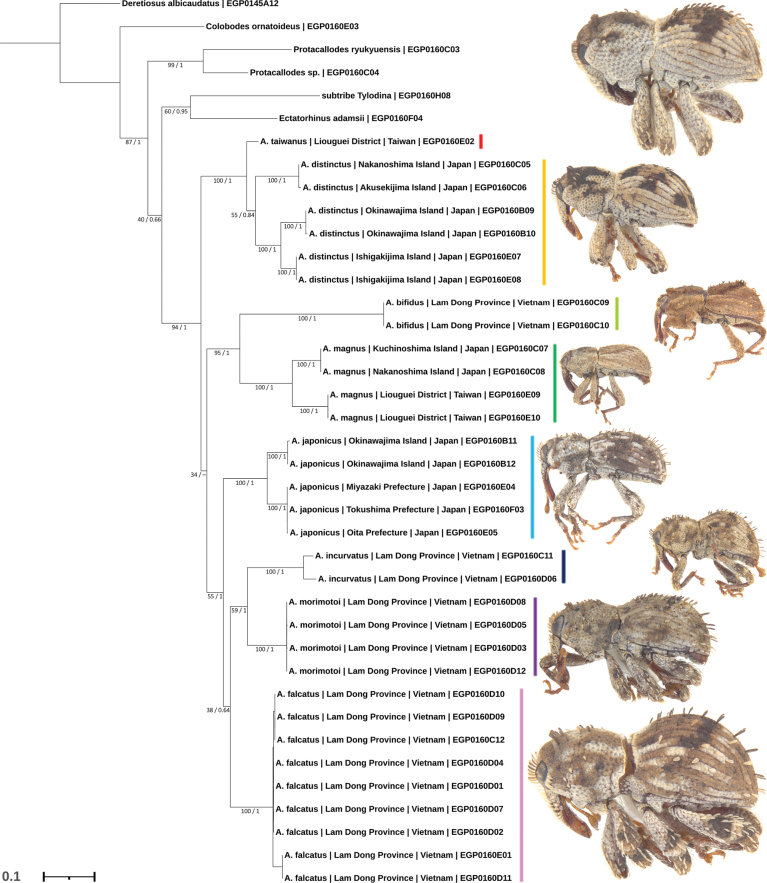
Maximum likelihood tree of *Aphanerostethus* species based on an 872 base-pair long concatenated DNA matrix (CO1, Cytb, 16S) with gene-wise partition modelling (CO1 / Cytb: GTR+F+I+G4; 16S: GTR+F+G4) constructed in IQ-TREE v. 1.6.12. Branch support values represent standard nonparametric bootstraps (1000 replicates) and posterior probabilities. The symbol “–” indicates a posterior probability less than 50 (i.e., collapsed nodes in the Bayesian Inference tree). EGP codes represent DNA extraction codes and serve also as unique specimen identifiers. Associated *Aphanerostethus* weevil figures on the right of the tree are not to scale. Note that, except for *A.distinctus* and *A.taiwanus* (see Comments under the *A.taiwanus* species profile), the monophyly of all *Aphanerostethus* species is strongly supported (BS: 100, PP: 1).

We found strong morphological and molecular evidence that warrants the description of twelve new species, namely, *A.armatus* sp. nov., *A.bifidus* sp. nov., *A.darlingi* sp. nov., *A.decoratus* sp. nov., *A.falcatus* sp. nov., *A.incurvatus* sp. nov., *A.japonicus* sp. nov., *A.magnus* sp. nov., *A.morimotoi* sp. nov., *A.nudus* sp. nov., *A.spinosus* sp. nov., and *A.taiwanus* sp. nov. from Japan, Taiwan, Vietnam, and Malaysia. Including the previously described *A.vannideki* Voss, 1957 and *A.distinctus* (Morimoto & Miyakawa, 1985), this brings the total number of known species of *Aphanerostethus* to fourteen. In addition to differences in the male genitalia, the species are also separable by external morphology. Important external characters include the presence (vs absence) of a prominent prosternal canal, the presence (vs absence) of ventral femoral teeth, patterning of erect elytral scales, the elytra bearing erect scales (vs recumbent scales), the shape of the male metatibial uncus, the presence (vs reduction) of the scutellum, overall size, and color. Sexual dimorphism in the metatibial uncus is particularly remarkable; females all have simple metatibial unci, whereas the metatibial unci in the males of some species are modified in shape and species-specific (see Figs 1, 2). With the exception of *A.taiwanus* and *A.distinctus*, the molecular results (Fig. 3) strongly support the monophyly of all the species analyzed (BS: 100, PP: 1). Although *A.taiwanus* was consistently placed in the same clade as *A.distinctus* (BS: 100, PP: 1) as expected, its presumed basal position (i.e., sister to *A.distinctus*) is only weakly supported (BS: 55, PP: 0.84; see the Comments section in *A.taiwanus* species profile). Notably, the close phylogenetic relationship of *A.magnus* and *A.bifidus* is strongly supported (BS: 95, PP: 1), a finding which was expected based on morphology.

### ﻿X-ray microtomography

#### ﻿Metatibial unci

Remarkable species-specific sexual dimorphism in the metatibial uncus occurs in seven of the fourteen *Aphanerostethus* species examined here, namely, *A.bifidus* (Y-shaped, bifid uncus; Fig. 1A–D), *A.falcatus* (sickle-shaped uncus; simple, but with lateral projection; Fig. 1I–L), *A.incurvatus* (incurved uncus; Fig. 1M–P), *A.magnus* (ear-shaped uncus; Fig. 2A–D), *A.morimotoi* (boot-shaped uncus; truncated at apex, but with lateral projection; Fig. 2E–H), *A.spinosus* (complex uncus shape; large C-shaped plate with apical inward-facing projection; Fig. 2I–L), and *A.nudus* (weak inward spiral-shaped uncus; Fig. 2M–P).

#### ﻿Pronotal morphology

**Figure 4.**
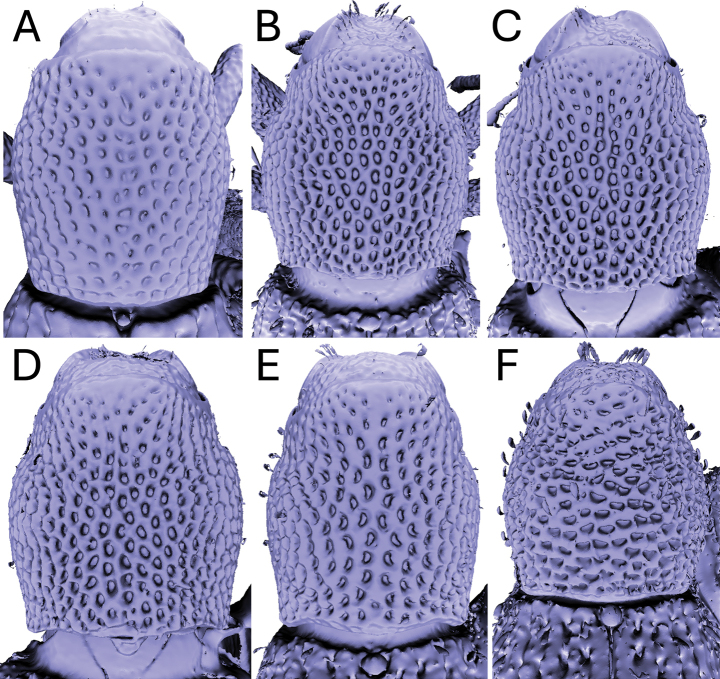
X-ray microtomography generated 3D models of *Aphanerostethus* pronota with scales removed, revealing otherwise hidden differences in underlying puncture morphology **A***Aphanerostethusarmatus* sp. nov. (JHLHY_DAR_078) **B–D***Aphanerostethusbifidus* sp. nov. (JHLHY_DAR_092, 101, and 103, respectively) **E***Aphanerostethusdecoratus* sp. nov. (JHLHY_DAR_079) **F***Aphanerostethusspinosus* sp. nov. (JHLHY_DAR_077).

**Figure 5.**
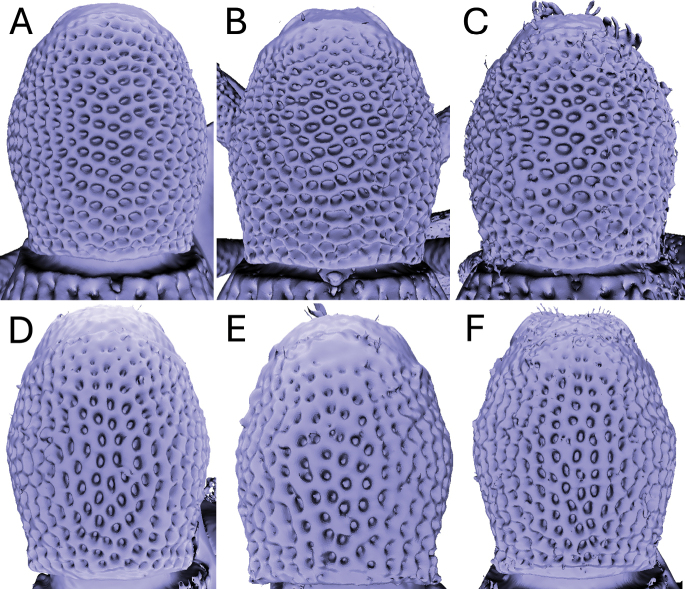
X-ray microtomography generated 3D models of *Aphanerostethus* pronota with scales removed, revealing otherwise hidden differences in underlying puncture morphology **A–C***Aphanerostethusdistinctus* (Morimoto & Miyakawa, 1985) (OKENT0089414, JHLHY_DAR_071, and 062, respectively) **D–F***Aphanerostethusfalcatus* sp. nov. (JHLHY_DAR_167, 170, and 172, respectively).

**Figure 6.**
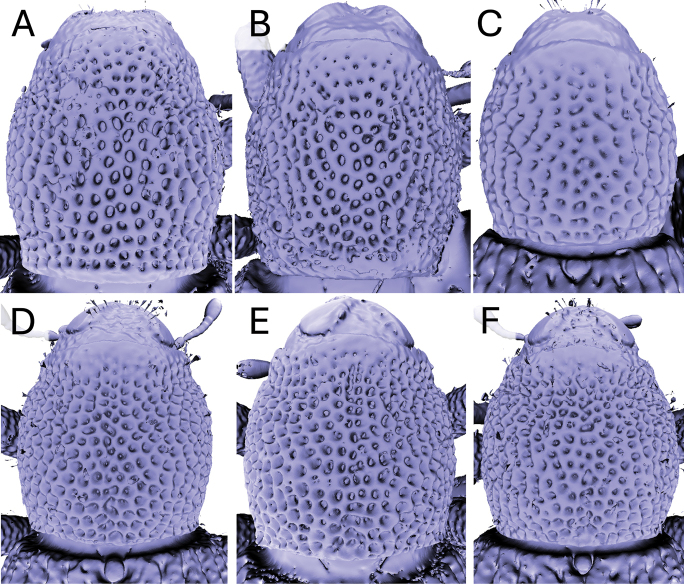
X-ray microtomography generated 3D models of *Aphanerostethus* pronota with scales removed, revealing otherwise hidden differences in underlying puncture morphology **A, B***Aphanerostethusincurvatus* sp. nov. (JHLHY_DAR_095, 104) **C–F***Aphanerostethusjaponicus* sp. nov. (OKENT0055232, JHLHY_DAR_051, 052, 041).

**Figure 7.**
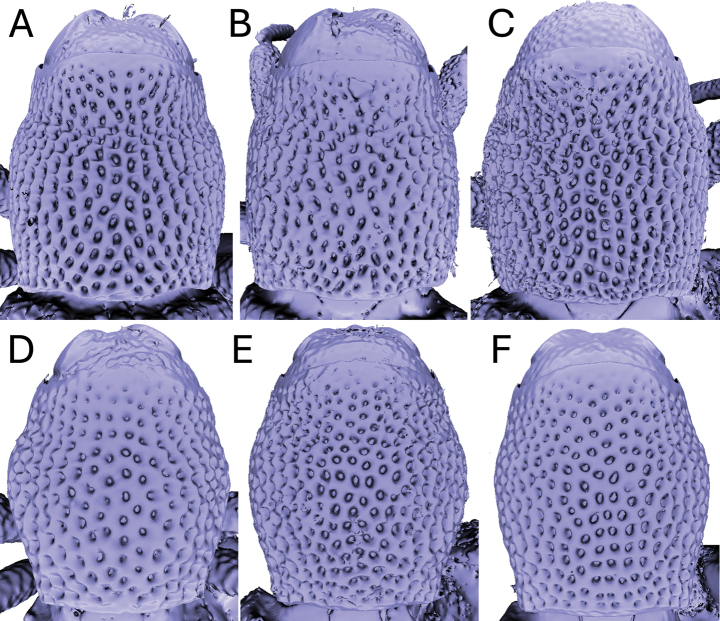
X-ray microtomography generated 3D models of *Aphanerostethus* pronota with scales removed, revealing otherwise hidden differences in underlying puncture morphology **A–C***Aphanerostethusmagnus* sp. nov. (JHLHY_DAR_029, 035, and 108, respectively) **D–F***Aphanerostethusmorimotoi* sp. nov. (JHLHY_DAR_189, 144, and 115, respectively).

**Figure 8.**
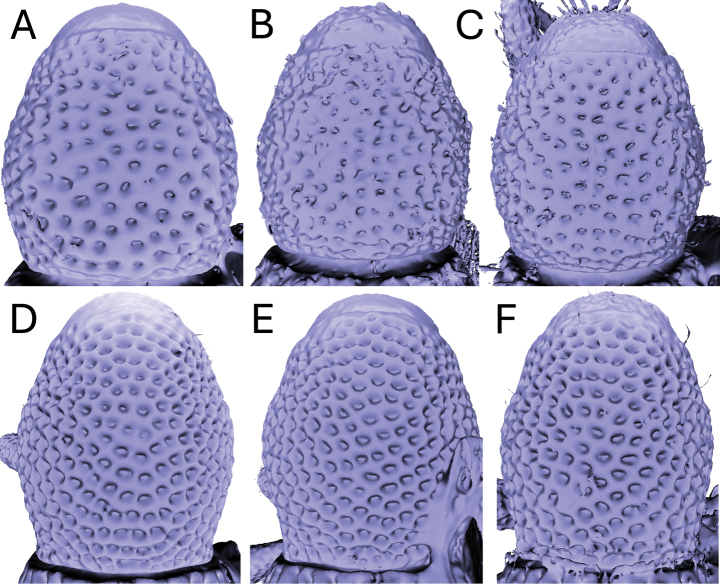
X-ray microtomography generated 3D models of *Aphanerostethus* pronota with scales removed, revealing otherwise hidden differences in underlying puncture morphology **A–C***Aphanerostethusnudus* sp. nov. (JHLHY_DAR_012, 013, and 014, respectively) **D–F***Aphanerostethustaiwanus* sp. nov. (JHLHY_DAR_070, 021, and 086, respectively).

**Figure 9.**
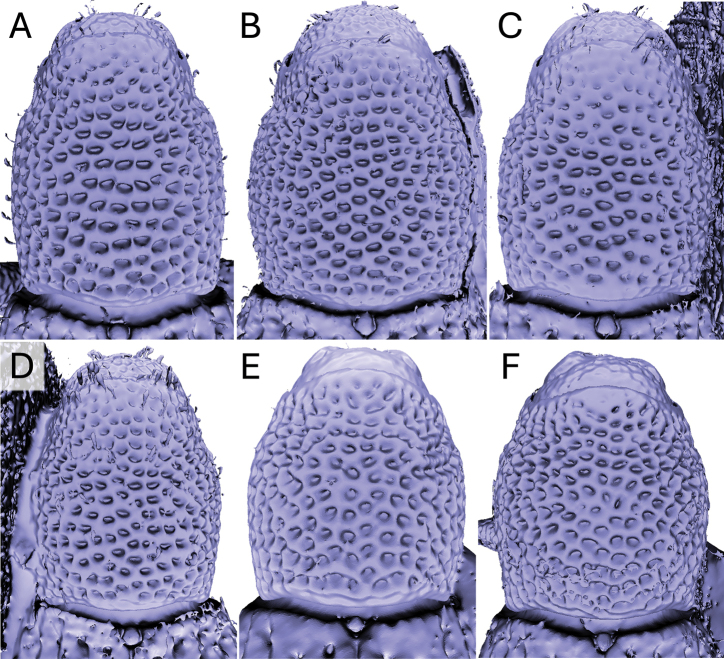
X-ray microtomography generated 3D models of *Aphanerostethus* pronota with scales removed, revealing otherwise hidden differences in underlying puncture morphology **A–D***Aphanerostethusvannideki* Voss, 1957 (JHLHY_DAR_082 (A), ZMA_5517696 (B), ZMH_841853 (C), and ZMA_5517698 (D), respectively) **E, F***Aphanerostethusdarlingi* sp. nov. (JHLHY_DAR_125, JHLHY_DAR_126).

Significant and stable interspecific differences in puncture shape and orientation were discovered after removing scales obscuring the dorsal region of the pronotum (in dorsal view; Figs 4–9). Punctures can be longitudinally elongate (*A.bifidus* (Fig. 4B–D), *A.magnus* (Fig. 7A–C)), C-shaped (*A.decoratus* (Fig. 4E), transversally elongate (*A.distinctus* (Fig. 5A–C), *A.spinosus* (Fig. 4F), *A.taiwanus* (Fig. 8D–F), *A.vannideki* (Fig. 9A–D), *A.darlingi* (Fig. 9E–F)), or approximately circular in shape (*A.armatus* (Fig. 4A), *A.falcatus* (Fig. 5D–F), *A.incurvatus* (Fig. 6A, B), *A.japonicus* (Fig. 6C–F), *A.morimotoi* (Fig. 7D–F), *A.nudus* (Fig. 8A–C)).

#### ﻿Tenth elytral stria

**Figure 10.**
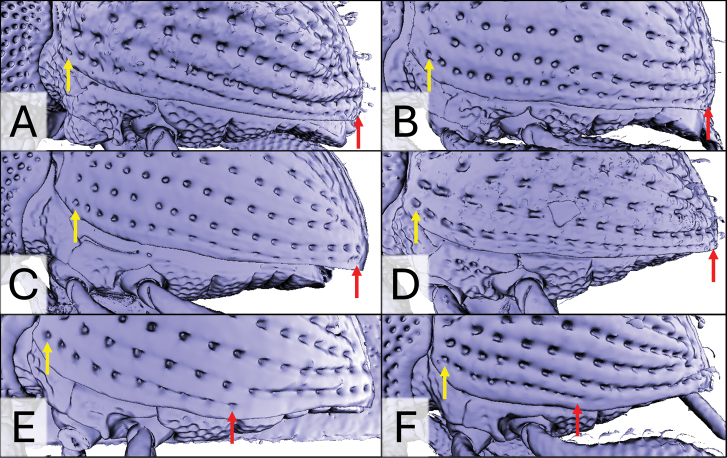
X-ray microtomography generated 3D models of *Aphanerostethus* elytra with scales removed, revealing differences in the length of the 10^th^ elytral stria. The yellow and red arrows indicate the base and apex of the 10^th^ elytral stria, respectively **A***Aphanerostethusbifidus* sp. nov. (JHLHY_DAR_092) **B***A.falcatus* sp. nov. (JHLHY_DAR_172) **C***A.morimotoi* sp. nov. (JHLHY_DAR_115) **D***A.decoratus* sp. nov. (JHLHY_DAR_079) **E***A.distinctus* (Morimotoi & Miyakawa, 1985) (OKENT0089414) **F***A.taiwanus* sp. nov. (JHLHY_DAR_070).

In most species (*A.bifidus*, *A.darlingi*, *A.decoratus*, *A.falcatus*, *A.incurvatus*, *A.japonicus*, *A.magnus*, *A.morimotoi*, *A.spinosus*) the tenth elytral stria extends from the base of the elytra to the apex, and includes 14–21 punctures (Fig. 10A–D). However, in *A.distinctus* and *A.taiwanus*, the tenth stria ends at the middle or slightly past the middle of the elytra, and includes ten punctures at most (Fig. 10E, F).

#### ﻿Hind wings

**Figure 11.**
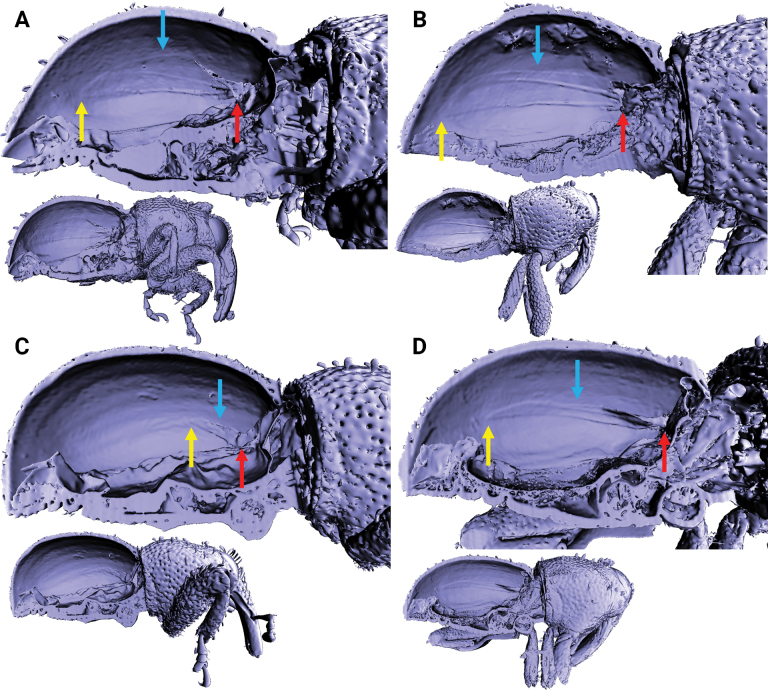
X-ray microtomography generated 3D models of *Aphanerostethus* with the right elytron removed, revealing differences in the length, width, and venation of the hindwing (as shown here, the hindwing is affixed to the inner surface of the elytron in most specimens). A lateral view of the full body is shown below each close-up for reference. The red, blue, and yellow arrows indicate the base, midpoint, and apex of the hindwing, respectively **A***Aphanerostethusbifidus* sp. nov. (JHLHY_DAR_092) **B***A.decoratus* sp. nov. (JHLHY_DAR_079) **C***A.japonicus* sp. nov. (JHLHY_DAR_041) **D***A.magnus* sp. nov. (JHLHY_DAR_032).

All known *Aphanerostethus* species are flightless; however, the amount of hindwing reduction in the genus varies interspecifically (Fig. 11). The degree of hindwing reduction can be classified into four categories: (1) only a minute stub of hindwing remains (*A.distinctus*, *A.taiwanus*, and *A.nudus*); (2) the hindwing remnants are elongate in shape but not reaching the middle of the elytra (*A.falcatus*, *A.japonicus* (Fig. 11C), *A.spinosus*); (3) the hindwing remnants are long, thin, and reaching past the middle of the elytra (*A.darlingi*, *A.incurvatus*, *A.magnus* (Fig. 11D), *A.morimotoi*, *A.vannideki*); (4) the hindwing remnants are long (reaching past the middle of the elytra), wide, and still clearly show the remains of longitudinal wing venation (*A.bifidus*, Fig. 11A; *A.decoratus*, Fig. 11B).

### ﻿Taxonomy

#### ﻿Genus Aphanerostethus

Taxon classificationAnimaliaColeopteraCurculionidae

Voss, 1957

2F3B530B-B558-5138-B235-2A2DDFF9B9CF

Darumazo Morimoto & Miyakawa, 1985, syn. nov.

##### Type species.

*Aphanerostethusvannideki* Voss, 1957, by monotypy.

##### Gender.

Masculine.

##### Redescription.

Body length 1.5–3.0 mm. Rounded to bulb-like appearance in lateral view. ***Cuticle***: Dark to pale red. ***Scalepattern***: Large flat white, gray, brown, or golden scales covering the body in most species (the cuticle of *A.nudus* is largely exposed); two prominent rows of erect scales (“eyelashes”) along inner margin of eye which extends ventrally to basal third to middle of rostrum; pronotum with erect or recumbent scales; erect or recumbent scales always on odd elytral intervals; erect scales on even elytral intervals in some species. ***Head***: Rostrum punctate, evenly curved; eyes large, ovate; antennae with six-articled funicle (except apparently in *A.armatus* which has five articles). ***Prothorax***: Densely punctate; margin of prothorax (in lateral view) extending forward, covering part of the eye (or most of it when rostrum fully set into prosternal sulcus); prosternum with strongly or weakly defined prosternal canal. ***Elytra***: Scutellum prominent or reduced; elytra rounded to bulb-like, with intervals convex; flightless, hind wings reduced to a stub or long filament. ***Abdomen***: Lateral edge of first abdominal segment contiguous with metanepisternum. ***Legs***: Femora with or without apico-ventral tooth; unci of fore- and mid-tibiae simple; unci of male hind-tibiae modified (bifid, hooked, or twisted) in some species, simple (evenly curved) in females; third tarsal segment bilobed; tarsal claws simple.

##### Distribution.

*Aphanerostethus* species are known from Japan (Izu Islands, mainland, Nansei Islands), Taiwan, Vietnam (Mt. Lang Biang), Malaysia (Cameron Highlands, Pahang; Sabah, Borneo), and Indonesia (West Java).

##### Notes.

The genus *Darumazo* Morimoto & Miyakawa, 1985 was described based on a single species, *D.distinctus* Morimoto & Miyakawa, 1985, from specimens collected in the Izu Islands, the Nansei Islands, and mainland Japan. Morimoto and Miyakawa (1985) distinguished *Darumazo* from *Aphanerostethus* as the latter have separated fore-coxae as well as toothed femora. However, the twelve new species described here reveal a continuum of forms, relating *A.distinctus* and *A.vannideki* with respect to the above two characters. In particular, although *A.vannideki* has separated fore-coxae and a longer prosternal canal, the related *A.bifidus* possesses weakly separate fore-coxae (contiguous in the related *A.magnus*). Furthermore, the femoral teeth are present but small in *A.morimotoi* (femoral teeth often completely absent) and *A.japonicus*, indicating continuity in this character across the genus as well. We do not feel that a moderate degree of separation in the fore-coxae, nor the presence (vs absence) of femoral teeth is enough to justify the continued recognition of *Darumazo*, especially in light of the continuity of forms newly described here. On the basis of these morphological considerations, we treat *Darumazo*, syn. nov. as a junior synonym of *Aphanerostethus*. The genus *Aphanerostethus* is currently placed in Cryptorhynchini Schoenherr, 1825 and *Darumazo* in Sthereini Hatch, 1971 (Alonso-Zarazaga and Lyal 1999; Alonso-Zarazaga et al. 2023). We are currently working on a molecular phylogeny of *Aphanerostethus* and some undescribed, related genera and hope that this reveals the tribal placement of the genus. Until that work is completed, we treat the genus as Molytinae incertae sedis.

###### ﻿*Aphanerostethus* species profiles

#### ﻿ Aphanerostethus armatus

Taxon classificationAnimaliaColeopteraCurculionidae

Lewis & Kojima sp. nov.

1A8C6424-B575-507D-A80D-73ECD3220A39

https://zoobank.org/6B31DB80-5F3E-48D6-82F1-1EAF13CBCE3B

**Figure 12.**
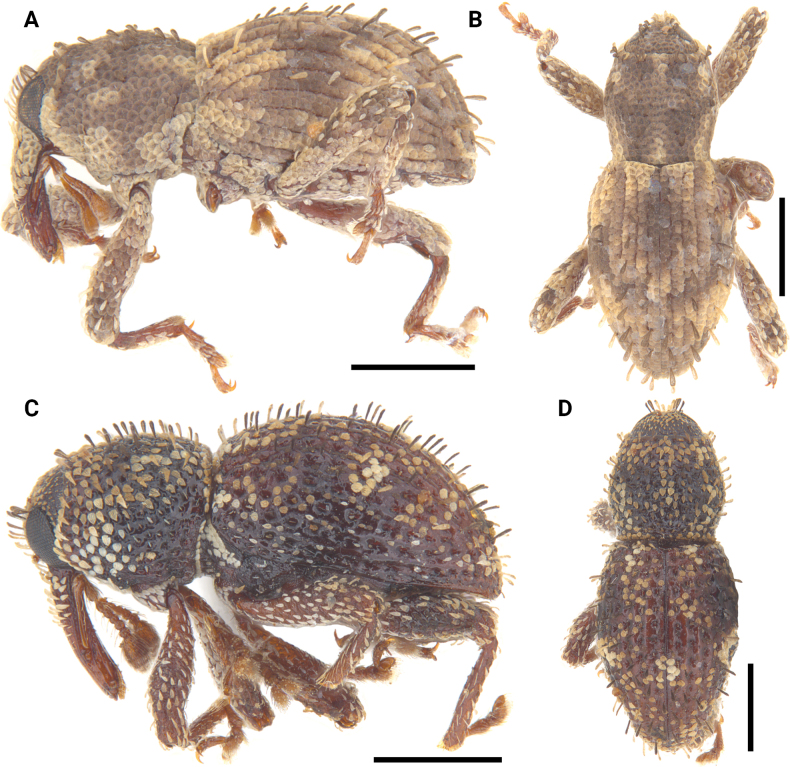
Lateral and dorsal photographs of *Aphanerostethus* species **A, B***Aphanerostethusarmatus* sp. nov. (JHLHY_DAR_078) **C, D***Aphanerostethusnudus* sp. nov. (JHLHY_DAR_014). Scale bars: 0.5 mm.

**Figure 13.**
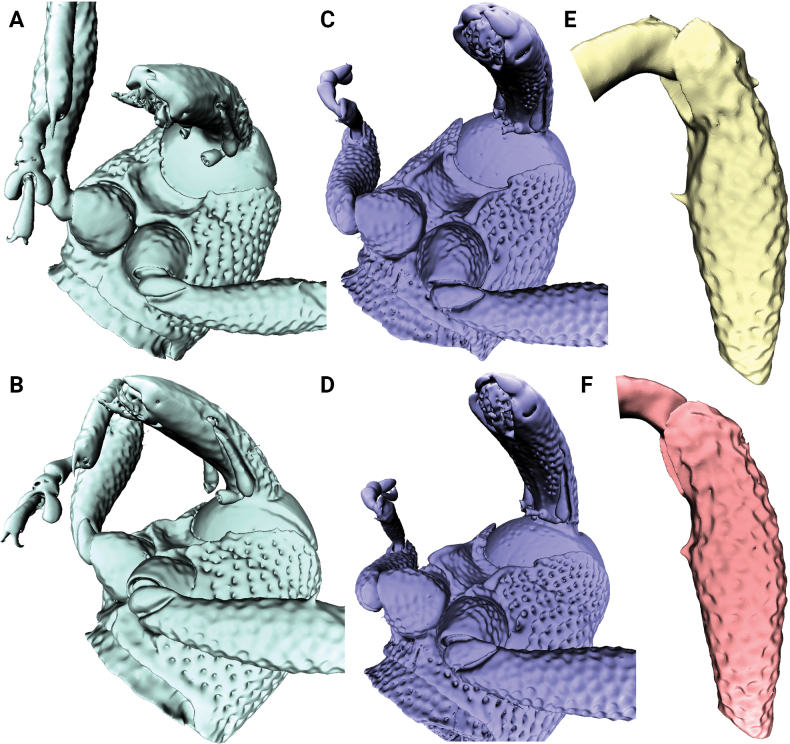
X-ray microtomography generated 3D models of *Aphanerostethus***A, B***A.armatus* sp. nov. (JHLHY_DAR_078) shallow prosternal canal **C, D***A.magnus* sp. nov. (JHLHY_DAR_029) deep prosternal canal **E***A.armatus* (JHLHY_DAR_078) fore-femur showing elongate, thorn-like ventral tooth **F***A.japonicus* sp. nov. (JHLHY_DAR_051) fore-femur showing blunt, obtuse ventral tooth.

Figs 4A , 12A, B , 13A, B, E

##### Specimens examined.

***Holotype*: Malaysia**: • Perak, Taiping, Bukit Larut, 7.I.1990, T. Yasunaga, deposited in KUM, JHLHY_DAR_078.

##### Diagnosis.

Body length 1.7 mm. Cuticle coated in crusty dark, sandy gray, and white scales in weakly defined pattern. Funicle with five articles. Only odd elytral intervals with erect scales. Prosternal cavity very weakly defined and without steep lateral ridges (Fig. 13A, B). Procoxae contiguous. Erect elytral scales evenly distributed, not concentrated in bundle. Scutellum not prominent. Femora each with elongate, thorn-like tooth ventrally (Fig. 13E). Metaventrite flattened between meta- and mesocoxae, without a distinct elevated transverse ridge.

##### Distribution.

This species is currently only known from Perak, Malaysia.

##### Etymology.

The specific name *armatus* is a Latin adjective that refers to the distinctly elongate, sharp tooth on the ventral edge of each femur (see Fig. 13E).

#### ﻿ Aphanerostethus bifidus

Taxon classificationAnimaliaColeopteraCurculionidae

Kojima & Lewis sp. nov.

683050BA-4C51-5D1A-97B0-0775394988E2

https://zoobank.org/F64611EA-ECDE-42DE-B1AF-C77ADC0B5A69

**Figure 14.**
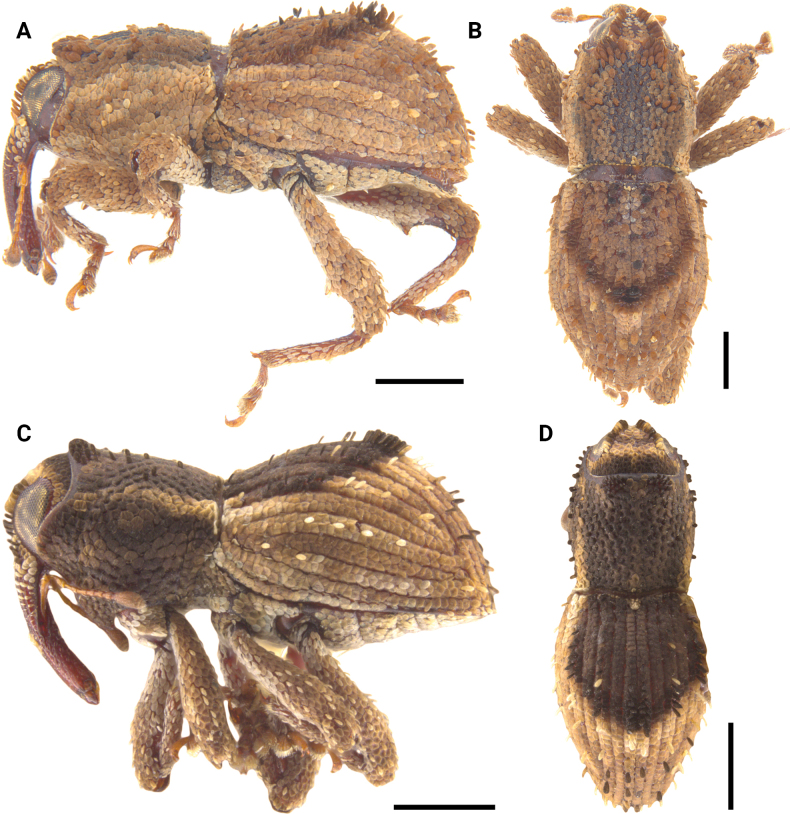
Lateral and dorsal photographs of *Aphanerostethus* species **A, B***Aphanerostethusbifidus* sp. nov. (JHLHY_DAR_092) **C, D***Aphanerostethusdecoratus* sp. nov. (JHLHY_DAR_079). Scale bars: 0.5 mm.

**Figure 15.**
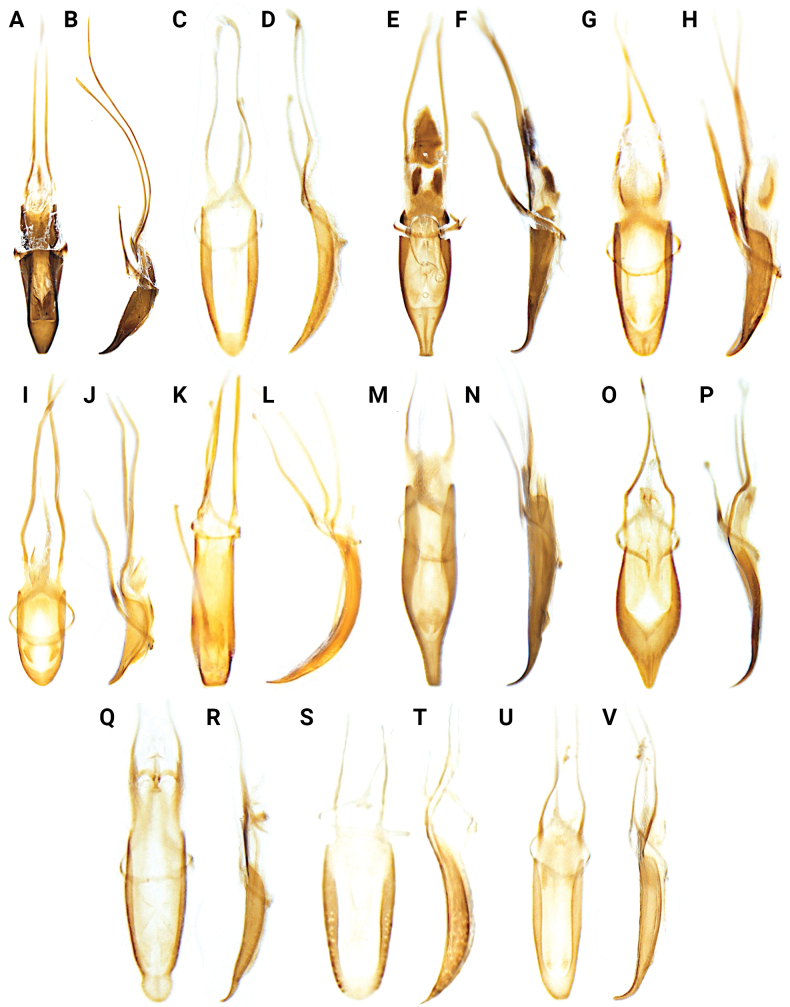
Aedeagi of *Aphanerostethus* species **A, B***A.bifidus* sp. nov. (JHLHY_DAR_102) **C, D***A.distinctus* (Morimoto & Miyakawa, 1985) (OKENT87658) **E, F***A.falcatus* sp. nov. (JHLHY_DAR_099) **G, H***A.incurvatus* sp. nov. (JHLHY_DAR_100) **I, J***A.japonicus* sp. nov. (JHLHY_DAR_052) **K, L***A.magnus* sp. nov. (JHLHY_DAR_022) **M, N***A.morimotoi* sp. nov. (JHLHY_DAR_113) **O, P***A.nudus* sp. nov. (JHLHY_DAR_012) **Q, R***A.spinosus* sp. nov. (JHLHY_DAR_077) **S, T***A.taiwanus* sp. nov. (JHLHY_DAR_016) **U, V***A.vannideki* Voss, 1957 (JHLHY_DAR_082).

Figs 1A–D , 3 , 4B–D , 10A , 11A , 14A, B , 15A, B

##### Specimens examined.

***Holotype*: Vietnam**: • Lam Dong Province, Mount Lang Biang, 12°02'N, 108°26'E, elevation 1700 m, 18.II.2011, H. Kojima, male deposited in TUA, JHLHY_DAR_092. ***Paratypes***: • Lam Dong Province, Mount Lang Biang, 12°02'N, 108°26'E, elevation 1700 m, 18.II.2011, H. Kojima, (1, TUA; 1, OIST), JHLHY_DAR_101 (EGP0160C09), JHLHY_DAR_102; • same locality, 26.II.2013, H. Kojima, (1, TUA), JHLHY_DAR_103 (EGP0160C10).

##### Diagnosis.

Body length 2.7–2.9 mm. Cuticle covered in dark to pale brown scales, with distinct dark, V-shaped band across anterior part of elytra. Funicle with six articles. Prosternal cavity prominent and with steep lateral ridges. Procoxae slightly separated. Second and odd-numbered elytral intervals with erect scales. Erect elytral scales concentrated in bundle on first, third, and fifth interval along V-shaped band. Scutellum prominent. Femora with large ventral tooth at midpoint. Metaventrite with a distinct elevated transverse ridge separating the meta- and mesocoxae. Metatibial uncus bifid in male (Fig. 1A–D). Aedeagus with distinctly long apodemes, and subquadrate in apical half (Fig. 15A, B).

##### Distribution.

This species is currently only known from Mount Lang Biang, Vietnam.

##### Etymology.

The specific name *bifidus* is a Latin adjective in reference to the bifid metatibial uncus observed in males (Fig. 1A–D).

#### ﻿ Aphanerostethus darlingi

Taxon classificationAnimaliaColeopteraCurculionidae

Lewis sp. nov.

B0E2D3FF-D5F1-5A30-81A4-43107F1CB7DE

https://zoobank.org/AF2C84EB-9B60-451A-8327-F6A76FE4DD8B

Figs 9E, F , 16A, B

##### Specimens examined.

***Holotype*: Malaysia: Borneo: Sarawak**: • Gunung Mulu National Park, 1387 m, Camp 3, 4°2.284'N, 114°53.36'E, 27.XI.2009–12.I.2010, malaise trap, D.C. Darling, B. Hubley, deposited in SFDK, ROM_OSU 308049, JHLHY_DAR_126. ***Paratype*: Malaysia: Borneo: Sarawak**: • Gunung Mulu National Park, 1387 m, Camp 3, 4°2.284'N, 114°53.36'E, 24.VII–21.IX.2011, malaise trap, D.C. Darling, (1, CMNC), ROM_OSU 308460, JHLHY_DAR_125.

**Figure 16.**
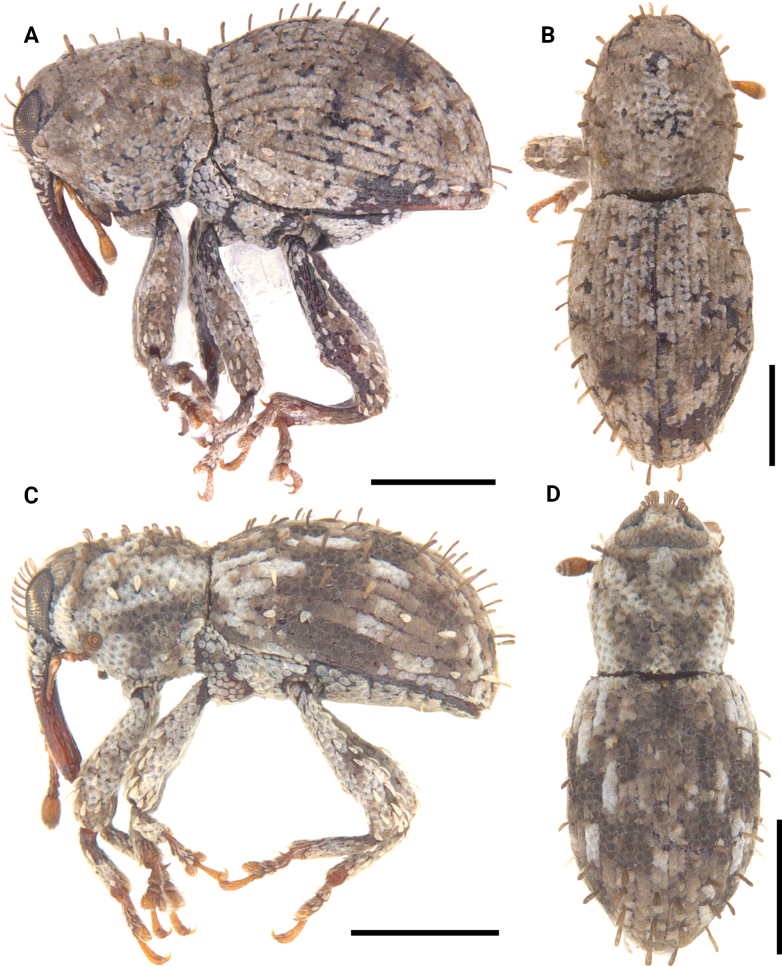
Lateral and dorsal photographs of *Aphanerostethus* species **A, B***A.darlingi* sp. nov. (JHLHY_DAR_126) **C, D***A.japonicus* sp. nov. (OKENT0055168). Scale bars: 0.5 mm.

##### Diagnosis.

Body length 2.0 mm. Cuticle coated in crusty dark, sandy gray, and white scales in weakly defined pattern. Funicle with six articles. Procoxae contiguous. Only odd-numbered elytral intervals with erect scales. Erect elytral scales evenly distributed, not concentrated in bundle. Scutellum not distinct. Femora each with prominent tooth. Prosternal cavity prominent and with steep lateral ridges. Metaventrite with small tubercle between meta- and mesocoxae, not a distinct transverse ridge.

##### Distribution.

This species is currently only known from Gunung Mulu National Park, Borneo, Malaysia.

##### Etymology.

The specific name *darlingi* honors the collector of the type series, Christopher Darling (Royal Ontario Museum), for his contributions to entomology in southeast Asia. It is a genitive, invariable.

#### ﻿ Aphanerostethus decoratus

Taxon classificationAnimaliaColeopteraCurculionidae

Lewis & Kojima sp. nov.

18A0BBAE-835B-5F66-AA3E-7B58AA20FAAD

https://zoobank.org/DDA220E8-BD2E-4EFE-998C-E0848B431F6B

Figs 4E , 10D , 11B , 14C, D

##### Specimens examined.

***Holotype*: Malaysia**: • Cameron Highlands, 2.IV.1990, J. Mateu, deposited in KUM, JHLHY_DAR_079.

##### Diagnosis.

Body length 2.2 mm. Cuticle covered in dark to pale brown scales, with distinct, dark, V-shaped band across anterior part of elytra. Funicle with six articles. Procoxae contiguous. Prosternal cavity prominent and with steep lateral ridges. Only odd-numbered elytral intervals with erect scales. Elytral scales concentrated in bundle on first interval at midpoint. Scutellum prominent. Femora with large ventral tooth at midpoint. Metaventrite with a distinct elevated transverse ridge separating the meta- and mesocoxae.

##### Distribution.

This species is currently only known from one specimen collected at Cameron Highlands, Malaysia.

##### Etymology.

The specific name *decoratus* is a Latin participle in reference to the posterior half of the elytra which is adorned with a dense tuft of erect scales.

#### ﻿ Aphanerostethus distinctus

Taxon classificationAnimaliaColeopteraCurculionidae

(Morimoto & Miyakawa, 1985) comb. nov.

B5076194-9EFD-5AD6-AEBE-7C26419F64EA

**Figure 17.**
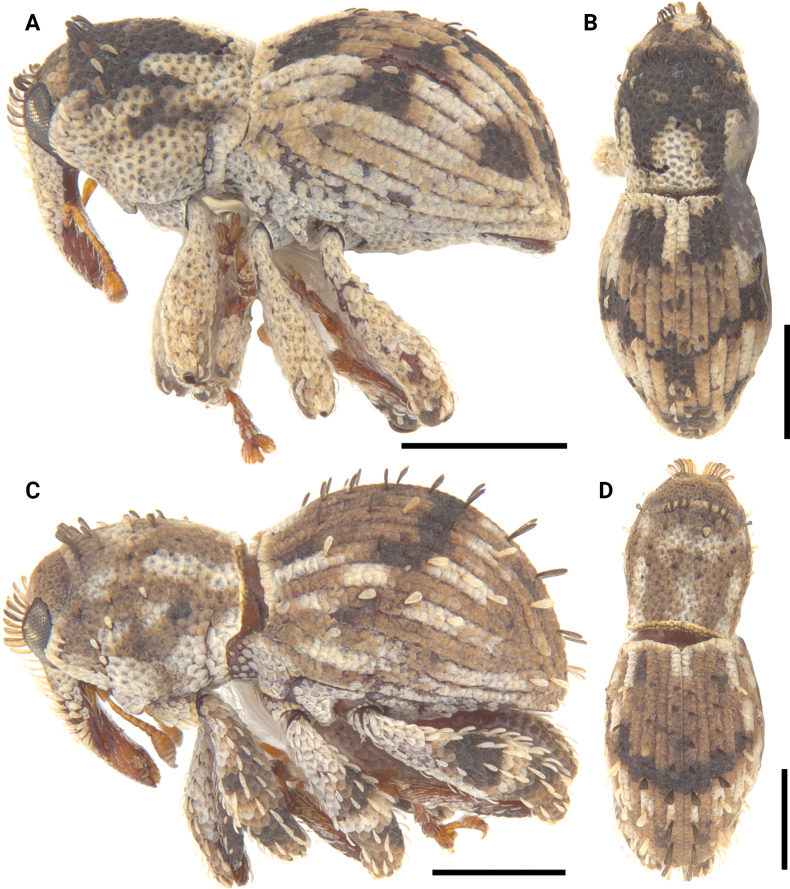
Lateral and dorsal photographs of *Aphanerostethus* species **A, B***Aphanerostethusdistinctus* (Morimoto & Miyakwa, 1985) (OKENT0087605) **C, D***Aphanerostethusfalcatus* sp. nov. (JHLHY_DAR_167). Scale bars: 0.5 mm.

**Figure 18:**
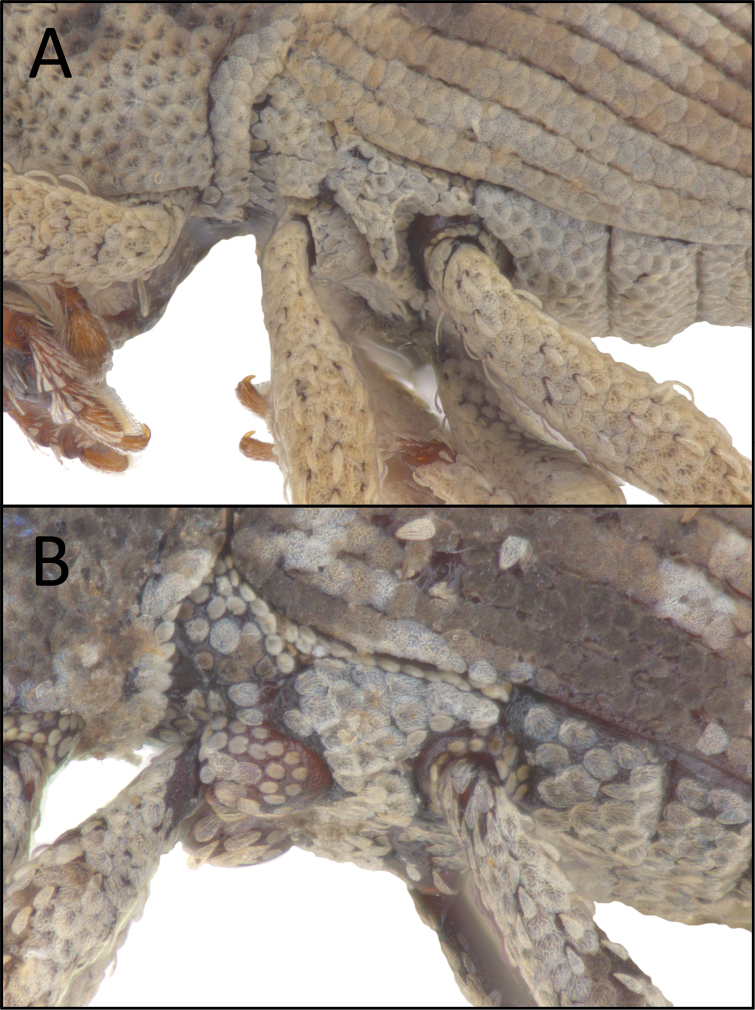
**A** Prominent ridge between meso- and metacoxae (*Aphanerostethusdistinctus* (Morimoto & Miyakawa, 1985), OKENT0087604) **B** No prominent ridge between meso- and metacoxae (*A.japonicus* sp. nov., OKENT0055168).

Figs 1E–H , 3 , 5A–C , 10E , 15C, D , 17A, B , 18A

Darumazo distinctus Morimoto & Miyakawa, 1985.

##### Specimens examined.

***Holotype*: Japan: Tokyo City**: • Miyake Island, Sabigahama, 2.V.1975, T. Mikage, deposited in ELKU, JHLHY_DAR_002. ***Paratypes*: Japan: Kagoshima Prefecture**: • Akuseki Is., 24.IV.1971, M. Sakai, (1, KUM), JHLHY_DAR_069; • **Nagasaki Prefecture**: Nagasaki City, Mt. Kompira, 25.III.1953, H. Kamiya, (1, KUM), JHLHY_DAR_003; • **Okinawa Prefecture**: Iriomote Is., Ushikumori, 9.III.1964, Y. Miyatake, (1, KUM), JHLHY_DAR_009; • Yonaguni Is., Sonai, 25–29.VIII.1969, H. Makihara, (1, KUM), JHLHY_DAR_010; • **Tokyo City**: Izu Oshima Is., Sashikiji, 3.V.1979, S. Miyakawa, (1, PCHY), JHLHY_DAR_004; • Izu Oshima Is., Mt. Atagoyama, 30.IV.1979, S. Miyakawa, (1, KUM), JHLHY_DAR_005; • Miyake Is., Sabigahama, 2.V.1975, T. Mikage, (1, KUM), JHLHY_DAR_006; • Aogashima Is., Ike no Sawa, 23.V.1979, J. Okuma, (1, KUM), JHLHY_DAR_007; • Hachijo-jima Is., Mihara rindo, 20.IV.1978, S. Miyakawa, (1, KUM), JHLHY_DAR_008; • Miyake Is., Nanto rindo, 27.IV.1978, Jun Okuma, (1, KUM), JHLHY_DAR_068.

##### Non-type material examined.

**Japan: Fukui Prefecture**: • Mikata-chou, Ongami Is., 5.X.1986, T. Ueno, (1, KUM), JHLHY_DAR_071, JHLHY_DAR_072. **Kagoshima Prefecture**: • Iwayadomari, Kuchinoerabu-jima Island, 28.VII.2013, H. Kojima, on *Boehmeria* sp., (50, TUA), JHLHY_DAR_200 – JHLHY_DAR_249; • Sato, Nakanoshima Is., 7–9.VII.2019, S. Imada, (1, KUM), JHLHY_DAR_067; • Yoriki, Nakanoshima Island, 15.III.2013, H. Kojima, (2, TUA), JHLHY_DAR_105 (EGP0160C05), JHLHY_DAR_143; • Mt. Otake, Nakanoshima Island, 13–16.III.2013, H. Kojima, (3, TUA), JHLHY_DAR_144; • Ookizaki, Nakanoshima Island, 30.IX.2015, H. Kojima, (1, TUA), JHLHY_DAR_145; • Mt. Negamiyama, Akuseki Island, 7–8.III.2013, H. Kojima, (2, TUA), JHLHY_DAR_146, JHLHY_DAR_147; • Mt. Birouyama, Akuseki Island, 6–8.III.2013, H. Kojima, (4, TUA), JHLHY_DAR_106 (EGP0160C06), JHLHY_DAR_148 – JHLHY_DAR_150; **Okinawa Prefecture**: • Kunigami, 20.IV.–12.VI.2006, H. Goto, (1, FFPRI), JHLHY_DAR_011; • Okinawa Is., Kunigami, Oku (26.83604°N, 128.27191°E), 27.V.–10.VI.2016, L. Iha, S. Iriyama, (1, OIST), OKENT0089219, EGP0160B10; • same locality, 8–22.VII.2016, Y. Tamaki, I. Maehira, (4, OIST), OKENT0089411–OKENT0089414, EGP0160B09; • Okinawa Is., Kunigami, Oku (26.83630°N, 128.27051°E), 8–22.VII.2016, Y. Tamaki, I. Maehira, (3, OIST), OKENT0087604–OKENT0087606; • same locality, 22.VII.–5.VIII.2016, T. Kinjo, K. Uekama, (1, OIST), OKENT0087637; • same locality, 5–19.VIII.2016, Y. Tamaki, K. Uekama, (1, OIST), OKENT0087658; • same locality, 19.VIII–2.IX.2016, K. Uekama, T. Yoshida, (1, OIST), OKENT0087677; • Iriomote Is., Funaura, 8.X.1977, S. Azuma, (1, RUMC), JHLHY_DAR_020; • Iheya Is., Koshi-dake, 4.V.1988, T. Ueno, (1, KUM), JHLHY_DAR_060; • Yonaguni Is., Mt. Urabu, 29.XII.1988, T. Ueno, (2, KUM), JHLHY_DAR_061, JHLHY_DAR_073; • Yonaguni Is., Mt. Kubura-dake, 26–31.III.1997, Tadashi Ishikawa, (1, PCHY), JHLHY_DAR_062; • same locality, 6.VII.1993, K. Shigematsu, (2, TUA), JHLHY_DAR_151, JHLHY_DAR_152; • Ishigaki Is., Nosko, Mt. Nosoko-dake, 24.4889°N, 124.2487°E, 16.III.2013, H. Yoshitake, (2, PCHY), JHLHY_DAR_063 (EGP0160E07), JHLDAR_DAR_064 (EGP0160E08); **Tokyo City**: • Hachijo-jima Is., 25.X.1962, R. Aoki, (1, KUM), JHLHY_DAR_128.

##### Diagnosis.

Body length 1.5–2.0 mm. Cuticle coated in dark, sandy gray, and white scales in contrasting pattern. Funicle with six articles. Procoxae contiguous. Only odd-numbered elytral intervals with recumbent scales. Erect elytral scales evenly distributed, not concentrated in bundle. Femora without ventral teeth. Scutellum reduced. Aedeagus short, with two sclerotized structures apico-laterally (one on each side) in internal sac. Prosternal cavity prominent and with steep lateral ridges. Metaventrite with a distinct elevated transverse ridge separating the meta- and mesocoxae (Fig. 18A). Metatibial uncus simple in both sexes (Fig. 1E–H). Aedeagus weakly tapering in apical half, and evenly curved in lateral view (Fig. 15C, D). Internal sac lacking prominent basal protruding structure (Fig. 15C, D).

##### Distribution.

This species is currently known from the Izu Islands (Miyake Is., Hachijo-jima Is., Aogashima Is.), Nagasaki Prefecture, Fukui Prefecture, Kagoshima Prefecture (Nakanoshima), and the Ryukyu Islands (Okinawa Is., Ishigaki Is., Yonaguni Is.) (Kojima and Morimoto 2004; Lewis and Yoshitake 2022). The *A.distinctus* records from Taiwan in Alonso-Zarazaga et al. (2023) and Kojima and Morimoto (2004) are more likely *A.taiwanus* as no specimens of *A.distinctus* were encountered in examined collections.

##### Comments.

Fujii et al. (2012) reared *A.distinctus* from the galls of *Asteralobiasasakii* (Monzen, 1937) on IlexcrenataThunb.var.hachijoensis Nakai, and Kojima (2013) reared specimens from the galls of *A.sasakii* on *Ilexintegra* Thunb. Kojima (2014) also collected 50 specimens from *Boehmeria* sp. on Kuchinoerabu-jima Island. On Okinawa Island, specimens of *A.distinctus* were only collected from Oku, Yambaru National Park (Lewis and Yoshitake 2022) despite several years of continuous, year-long malaise trapping at 24 sites (3 traps per site) across the island. This suggests that *A.distinctus* is sensitive to anthropogenic disturbance and, coupled with the apparent cecidophagous habits of the species, may be the reason for its relative rarity in collections.

#### ﻿ Aphanerostethus falcatus

Taxon classificationAnimaliaColeopteraCurculionidae

Kojima, Lewis & Fujisawa sp. nov.

FD7C6E27-C2A2-5232-9C38-3D168D564843

https://zoobank.org/8C985A03-E302-41EA-9754-B9F58E9B0E01

Figs 1I–L , 3 , 5D–F , 10B , 15E, F , 17C, D

##### Specimens examined.

***Holotype*: Vietnam: Lam Dong Province**: • Mount Lang Biang, 12°02'N, 108°26'E, elevation 1700 m, 18.II.2011, H. Kojima, male deposited in TUA, JHLHY_DAR_153. ***Paratypes*: Vietnam: Lam Dong Province**: • Mount Lang Biang, 12°02'N, 108°26'E, elevation 1700 m, 17–18.II.2011, H. Kojima, (21, TUA; 1 OIST), JHLHY_DAR_094, JHLHY_DAR_096 (EGP0160D01), JHLHY_DAR_097, JHLHY_DAR_099, JHLHY_DAR_110 (EGP0160C12), JHLHY_DAR_112 (EGP0160D02), JHLHY_DAR_116 (EGP0160D10), JHLHY_DAR_117 (EGP0160D09), JHLHY_DAR_119 (EGP0160D07), JHLHY_DAR_154 – JHLHY_DAR_166; • near Phi Lieng, Lam Ha, 21.II.2011, H. Kojima, (1, TUA), JHLHY_DAR_118 (EGP0160D11); • Da Knang, Dam Rong District, 23.II.2013, H. Kojima, (1, TUA), JHLHY_DAR_122 (EGP0160E01); • same locality, 2.III.2014, Y. Fujisawa, (1, TUA); • Mount Lang Biang, 12°02'N, 108°26'E, elevation 1700 m, 20–26.II.2013, H. Kojima, (18, TUA; 2, OIST), JHLHY_DAR_091, JHLHY_DAR_109 (EGP0160D04), JHLHY_DAR_168 – JHLHY_DAR_179, JHLHY_DAR_181 – JHLHY_DAR_186.

##### Diagnosis.

Body length 1.7–2.1 mm. Cuticle coated in dark, sandy gray, and white scales in indistinct pattern. Funicle with six articles. Procoxae contiguous. Only odd-numbered elytral intervals with erect scales. Erect elytral scales evenly distributed, not concentrated in bundle. Elytral interval 5 + 6 not distinctly arched at base. Hind femora with distinct tooth along ventral edge. Fore- and mid-femur with minute tooth. Scutellum reduced. Prosternal cavity prominent and with steep lateral ridges. Metaventrite with a distinct elevated transverse ridge separating the meta- and mesocoxae. Metatibial uncus sickle-shaped in male (Fig. 1I–L). Aedeagus tapering over apical half, and swelling at tip (Fig. 15E, F), unevenly curved in lateral view; clearly bent ventrally at apex (Fig. 15E, F). Internal sac with basal protruding structure (Fig. 15E, F).

##### Distribution.

This species is currently only known from Lam Dong Province (Mt. Lang Biang, Phi Lieng, and Da Knang), Vietnam.

##### Etymology.

The specific name *falcatus* is a Latin adjective in reference to the sickle-shaped metatibial uncus of males (Fig. 1I–L).

#### ﻿ Aphanerostethus incurvatus

Taxon classificationAnimaliaColeopteraCurculionidae

Kojima & Lewis sp. nov.

4F32033B-AB30-5CA3-A5FD-B44929AB9F63

https://zoobank.org/D8E5DE1D-E198-4A13-99CC-4BBFDE3395BF

**Figure 19.**
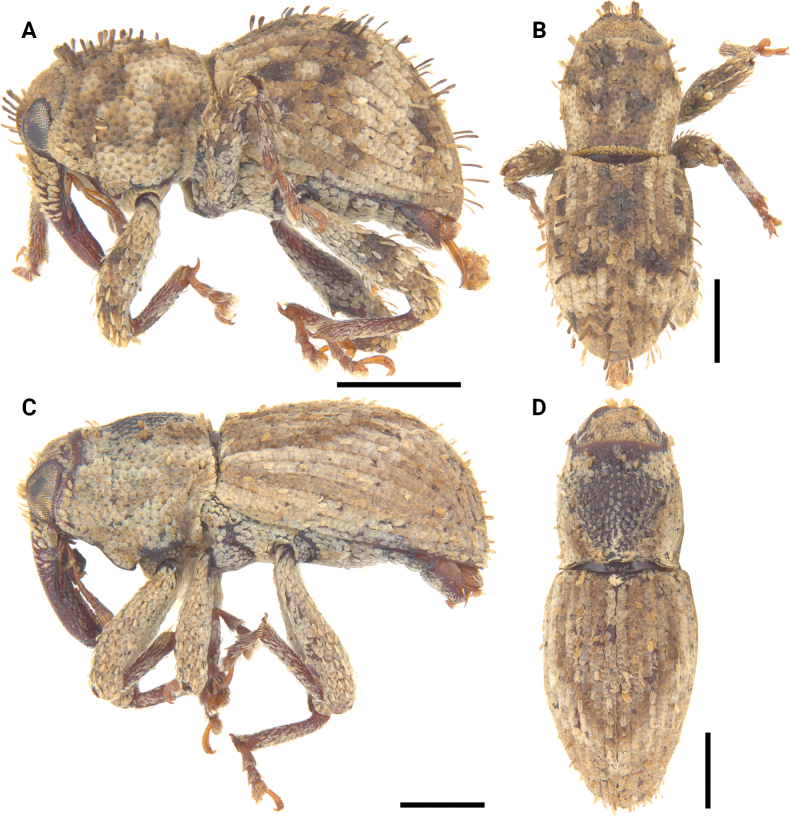
Lateral and dorsal photographs of *Aphanerostethus* species **A, B***Aphanerostethusincurvatus* sp. nov. (JHLHY_DAR_095) **C, D***Aphanerostethusmagnus* sp. nov. (JHLHY_DAR_023). Scale bars: 0.5 mm.

Figs 1M–P , 3 , 6A, B , 15G, H , 19A, B

##### Specimens examined.

***Holotype*: Vietnam**: • Lam Dong Province, Mount Lang Biang, 12°02'N, 108°26'E, elevation 1700 m, 17.II.2011, H. Kojima, male deposited in TUA, JHLHY_DAR_095. ***Paratypes*: Vietnam**: • Lam Dong Province, Mount Lang Biang, 12°02'N, 108°26'E, elevation 1700 m, 17.II.2011, H. Kojima, (1, TUA), JHLHY_DAR_104 (EGP0160C11); • same locality, 17.II.2011, H. Kojima, (1, TUA), JHLHY_DAR_100 (EGP0160D06).

##### Diagnosis.

Body length 1.7–2.1 mm. Cuticle coated in dark, sandy gray, and white scales in indistinct pattern. Funicle with six articles. Procoxae contiguous. Only odd-numbered elytral intervals with erect scales. Erect elytral scales concentrated in bundle along third elytral interval at midpoint; evenly distributed along other intervals. Elytral interval 5 + 6 distinctly arched at base. Femora all with large tooth along ventral edge. Scutellum reduced. Prosternal cavity prominent and with steep lateral ridges. Metaventrite with a distinct elevated transverse ridge separating the meta- and mesocoxae. Metatibial uncus curved inwards, and with minute black tubercles at base in males (Fig. 1M–P). Aedeagus evenly curved in lateral half, and in lateral view (Fig. 15G, H). Internal sac with U-shaped, basal protruding structure (Fig. 15G, H).

##### Distribution.

This species is currently only known from Mount Liang Bang, Lam Dong Province, Vietnam.

##### Etymology.

The specific name *incurvatus* is a Latin participle in reference to the incurved metatibial uncus of males (Fig. 1M–P).

#### ﻿ Aphanerostethus japonicus

Taxon classificationAnimaliaColeopteraCurculionidae

Lewis & Kojima sp. nov.

95FAB99E-9357-54EC-842C-ED6A899D09E0

https://zoobank.org/48E49909-06AB-42CD-AEC0-F3D68AE8D359

Figs 3 , 6C–F , 11C , 13F , 15I, J , 16C, D , 18B

##### Specimens examined.

***Holotype*: Japan: Miyazaki Prefecture**: • Gokase-chou, Shiraiwa-yama, 6.V.2009, Y. Tsutsumiuchi, male deposited in KUM, JHLHY_DAR_052. ***Paratypes*: Japan: Ehime Prefecture**: • Odamiyama, 2.IV.1986, E. Yamamoto, (1, KUM), JHL_SYN_197; **Kagawa Prefecture**: • Shionoe-chou, Ootaki-yama, 11.VII.1992, K. Kume, (1, KUM), JHLHY_DAR_053; **Miyazaki Prefecture**: • Gokase-chou, Shiraiwa-yama, 6.V.2009, Y. Tsutsumiuchi, (3, KUM; 1, OIST), JHLHY_DAR_048 – JHLHY_DAR_051; • Gokase, Mt. Mokouzaka, 17.VI.2017, R. Ito, (4, KUM), JHLHY_DAR_059 (EGP0160E04), JHL_SYN_194 – JHL_SYN_196; • Mt. Goyodake, Hinokage, 15.VII.2018, R. Ito, (1, KUM), JHL_SYN_199; **Oita Prefecture**: • Saiki-shi, Fujigawachi-keikoku, 10.IX.2017, R. Ito, (2, KUM), JHLHY_DAR_054, JHLHY_DAR_055; • Saiki-shi, Fujigawachi-keikoku, 18.V.2018, R. Ito, (3, KUM), JHLHY_DAR_056 – JHLHY_DAR_058, EGP0160E05; **Okinawa Prefecture**: • Okinawa Island, Kunigami, Yona Field (26.73894°N, 128.23720°E), 10–24.VI.2016, L. Iha, T. Kinjo, (1, OIST), OKENT0062435; • Okinawa Island, Kunigami, Yona Field (26.73894°N, 128.23720°E), 5–19.II.2016, L. Iha, T. Kinjo, (1, OIST), OKENT0055232 (EGP0160B12); • Okinawa Island, Kunigami, Yona Field (26.73894°N, 128.23720°E), 22.VII.–5.VIII.2016, T. Kinjo, K. Uekama, (1, OIST), OKENT0055516 (EGP0160B11); • Okinawa Island, Kunigami, Yona Field (26.73894°N, 128.23720°E), 27.XI.–11.XII.2015, Y. Tamaki, S. Iriyama, T. Yoshida, (1, OIST), OKENT0055168; **Tokushima Prefecture**: • Mima-gun, Tsurugi-chou, Ichiu, 33°53'N, 134°4'E, 31.VII.2011, K. Kume, (4, KUM; 1, OIST), JHLHY_DAR_037 – JHLHY_DAR_041; • Mt. Tomaru, Tsurugi-chou, 27.VII.2014, K. Kanno, (1, KUM), JHL_SYN_198; • Nishiiyayama-son, 33°58'N, 133°4'E, 4.VIII.2021, K. Kume, (1, KUM), JHLHY_DAR_042; • Miyoshi-shi, Mikamo-chou, Furonto, 22.VII.2012, K. Kume, (4, KUM; 1, OIST), JHLHY_DAR_043 – JHLHY_DAR_047 (EGP0160F03).

##### Diagnosis.

Body length 1.4–1.7 mm. Cuticle coated in crusty dark, sandy gray, and white scales in weakly defined pattern. Funicle with six articles. Procoxae contiguous. Eyes not dimorphic (separated in both sexes). Only odd-numbered elytral intervals with erect scales. Erect elytral scales evenly distributed, not concentrated in bundle. Scutellum distinct. Femora each with low, obtuse tooth ventrally. Prosternal cavity very weakly defined and without steep lateral ridges. Metaventrite flattened between meta- and mesocoxae, without a distinct elevated transverse ridge. Metatibial uncus simple in both sexes. Aedeagus rounded in lateral half (Fig. 15I, J). Internal sac with basal protruding structure (Fig. 15I, –J).

##### Distribution.

This species is currently only known from Japan, north from Tokushima Prefecture and Kagawa Prefecture, and in the Ryukyu Islands from Okinawa Prefecture (Yambaru National Park).

##### Etymology.

The specific name *japonicus* is a Latin adjective in reference to the country of collection. We also suggest the Japanese common name ニッポンダルマクチカクシゾウムシ [Nippon-daruma-kuchi-kakushi-zômushi], which translates in English to “Japanese daruma cryptorhynchine weevil”.

##### Variation.

Specimens of *A.japonicus* from Okinawa Island are noticeably slenderer than those collected on the mainland, but are otherwise indistinguishable and do not possess any structural characters that would support treating these as different species.

##### Comments.

The exact phylogenetic relationship of *A.japonicus* (along with *A.nudus* and *A.armatus*) to typical *Aphanerostethus* (i.e., those possessing a prominent prosternal canal and ridge between the meso- and metacoxae) remains uncertain given that the molecular data presented here does not clearly suggest whether they represent a lineage nested within or sister to typical *Aphanerostethus* (see Fig. 3). However, the fact that *A.nudus* possesses dimorphic metatibial uncus, a character which occurs sparsely in weevils, is significant and further supports the association of typical *Aphanerostethus* and the *A.nudus* group.

Like *A.distinctus* (see Comments under *A.distinctus*), this species was only collected from one site (Yona, Yambaru National Park) on Okinawa Island despite several years of malaise trapping at twenty-four sites across the island suggesting that it is also sensitive to anthropogenic disturbance.

#### ﻿ Aphanerostethus magnus

Taxon classificationAnimaliaColeopteraCurculionidae

Lewis & Kojima sp. nov.

2FF89F7D-89D8-5C52-8183-D219138E8B79

https://zoobank.org/CD1F7D2D-F57F-4C51-A6F3-9D971B75A03C

Figs 2A–D , 3 , 7A–C , 11D , 13C, D , 15K, L , 19C, D

##### Specimens examined.

***Holotype*: Japan: Kagoshima Prefecture**: • Nakanoshima Is., 1–2.V.1975, H. Irie, male deposited in KUM, JHLHY_DAR_023. ***Paratypes*: Japan: Kagoshima Prefecture**: • Nakanoshima Is., 1–2.V.1975, H. Irie, (4, KUM; 1, OIST), JHLHY_DAR_022, JHLHY_DAR_024 – JHLHY_DAR_026, JHLHY_DAR_076; • same locality, 7.VII.1974, J. Okuma, (2, KUM), JHLHY_DAR_027, JHLHY_DAR_028; • same locality, 5.VIII.1989, T. Ueno, (2, KUM; 1, OIST), JHLHY_DAR_029–JHLHY_DAR_031; • same locality, 28.V.1962, M. Sato, (1, KUM), JHLHY_DAR_034; • Nakanoshima, Sato, 7.VII.1974, J. Okuma, (1, KUM), JHLHY_DAR_032; • Nakanoshima, Satsuda, 14.VII.1982, Y. Takai, (1, KUM), JHLHY_DAR_033; • Nakanoshima, 29.IV.1987, S. Nomura, (1, KUM), JHLHY_DAR_075; • Nakanoshima, 14.VII.1986, H. Fujita, (1, HUM), JHLHY_DAR_141; • Nakanoshima, 6.VI.1953, (1, HUM), JHLHY_DAR_142; • Nakanoshima, 21.VII.1969, M. Sakai, (2, KUM), JHLHY_DAR_139, JHLHY_DAR_140; • Nakanoshima, entrance of Mt. Otake-tozandoro, 2.X.2015, H. Kojima, (1, TUA), JHLHY_DAR_108, EGP0160C08; • Kuchinoshima, Seranma, 5.V.2013, H. Kojima, (1, TUA), JHLHY_DAR_107, EGP0160C07; **Kouchi Prefecture**: • Okinoshima Is., 31.VII.1953, K. Morimoto, (1, KUM), JHLHY_DAR_035; **Taiwan: Kaohsiung City**: • Liouguei District, Zhong-Xing-Long Li, near Mt. Taiyuanshan, 19.X.2015, H. Yoshitake, (2, NMNST), JHLHY_DAR_065 (EGP0160E09), JHLHY_DAR_066 (EGP0160E10).

##### Diagnosis.

Body length 2.6–3.0 mm. Cuticle covered in dark to pale brown scales, with dark, V-shaped band across anterior part of elytra. Procoxae contiguous. Funicle with six articles. Second and odd-numbered elytral intervals with erect scales. Erect elytral scales variably concentrated in bundle on first elytral interval at apex of V-shaped band. Scutellum prominent and bulging. Elytral intervals moderately convex. Femora all with ventral tooth along ventral edge at midpoint. Prosternal cavity prominent and with steep lateral ridges. Metaventrite with a distinct elevated transverse ridge separating the meta- and mesocoxae. Metatibial uncus C-shaped in male (Fig. 2A–D). Aedeagus tapering in lateral half and weakly subquadrate at apex (Fig. 15K, L). Internal sac lacking prominent basal protruding structure (Fig. 15K, L).

##### Distribution.

This species is known from Nakanoshima Is. (Kagoshima Prefecture) and Okinoshima Is. (Kouchi Prefecture), Japan, as well as Zhong-Xing-Long Li (Liouguei District), Taiwan.

##### Etymology.

The specific name *magnus* is a Latin adjective in reference to the distinctly large body size and elongate aedeagus of this species. We suggest the Japanese common name オオダルマクチカクシゾウムシ [Oo-daruma-kuchi-kakushi-zômushi], which translates in English to “Big daruma cryptorhynchine weevil”.

##### Comments.

This species is closely allied with *A.bifidus*, a phylogenetic hypothesis which is also strongly supported by our molecular phylogenetic analysis (BS: 95, PP: 1). Both *A.bifidus* and *A.magnus* exhibit the same distinctive brown scaling pattern, similarity in metatibial uncus morphology, and general appearance. *Aphanerostethusmagnus* is present in the Osumi and Tokara Islands and Taiwan, but apparently absent from the more southern Nansei Island groups such as the Amami Islands, the Okinawa Islands, and the Sakishima Islands. This peculiar distributional pattern occurs in a number of other weevil species, such as *Acicnemissauteri* Hubenthal, 1917, *Dendropemonjaponicus* (Morimoto, 1979), *Orychodesplanicollis* (Walker, 1859), and *Stiboderesimpressus* (Jordan, 1912) (Kojima and Morimoto 2004), and possibly is explained by climatic and floristic differences between these regions as there are few large mountains in the Nansei Islands south of the Tokara Islands that could harbor high-altitude or more northerly distributed species.

#### ﻿ Aphanerostethus morimotoi

Taxon classificationAnimaliaColeopteraCurculionidae

Kojima & Lewis sp. nov.

65F18F99-CFBF-5130-B7AB-CC5DF5176AEF

https://zoobank.org/CA175CD9-FD04-45A7-A32C-3B71D07D11C0

**Figure 20.**
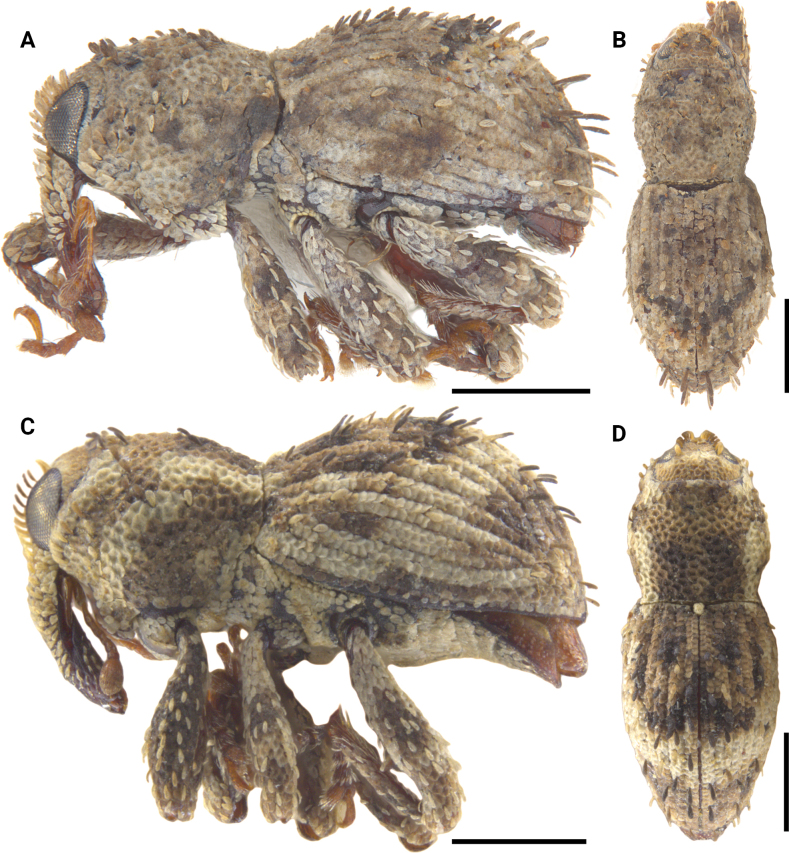
Lateral and dorsal photographs of *Aphanerostethus* species **A, B***Aphanerostethusmorimotoi* sp. nov. (JHLHY_DAR_093) **C, D***Aphanerostethusspinosus* sp. nov. (JHLHY_DAR_077). Scale bars: 0.5 mm.

Figs 2E–H , 3 , 7D–F , 10C , 15M, N , 20A, B

##### Specimens examined.

***Holotype*: Vietnam**: • Lam Dong Province, Mount Lang Biang, 12°02'N, 108°26'E, elevation 1700 m, 27.II.2011, H. Kojima, male deposited in TUA, JHLHY_DAR_093. ***Paratypes*: Vietnam**: • Lam Dong Province, Mount Lang Biang, elevation 1640–2000 m, 21–27.II.2013, H. Kojima, (2, TUA; 1, OIST; 1, KUM), JHLHY_DAR_113 – JHLHY_DAR_115 (EGP0160D03, EGP0160D08, EGP0160D12), JHLHY_DAR_187; • Lam Dong Province, Mount Lang Biang, 12°02'N, 108°26'E, elevation 1700 m, 17–18.II.2011, H. Kojima, (3, TUA; 1, OIST), JHLHY_DAR_111 (EGP0160D05), JHLHY_DAR_188 – JHLHY_DAR_190.

##### Diagnosis.

Body length 1.8–2.0 mm. Cuticle coated in dark, sandy gray, and white scales in indistinct pattern. Funicle with six articles. Procoxae contiguous. Only odd-numbered elytral intervals with erect scales. Erect elytral scales evenly distributed along intervals. Elytral interval 5 + 6 not distinctly arched at base. Femora with or without extremely minute tubercle or tooth along ventral edge. Scutellum reduced. Prosternal cavity prominent and with steep lateral ridges. Metaventrite with a distinct elevated transverse ridge separating the meta- and mesocoxae. Metatibial uncus in male curved inwards, and with minute black tubercles at base (Fig. 2E–H). Aedeagus sinuate near midpoint, and tapering prominently in lateral half (Fig. 15M, N), unevenly curved, but rather bent ventrally at apex (Fig. 15M, N). Internal sac with roughened basal protruding structure (Fig. 15M, N).

##### Distribution.

This species is currently only known from Mount Liang Bang, Lam Dong Province, Vietnam.

##### Etymology.

This eponym is dedicated to the late Dr. Katsura Morimoto, who pioneered the field of weevil taxonomy and amassed an invaluable specimen collection (at KUM) which we humbly make use of in this study.

#### ﻿ Aphanerostethus nudus

Taxon classificationAnimaliaColeopteraCurculionidae

Lewis & Kojima sp. nov.

9DF2B2A7-05A9-520F-BC44-C37C9860031F

https://zoobank.org/009FEE4B-6E20-47AD-AA5E-F8FDC817F490

Figs 2M–P , 8A–C , 12C, D , 15O, P

##### Specimens examined.

***Holotype*: Malaysia: Brinchang**: • 17.V.1973, S. Miyakawa, male deposited in KUM, JHLHY_DAR_014. ***Paratypes*: Malaysia: Brinchang**: • 17.V.1973, S. Miyakawa, (3, KUM; 1, OIST), JHLHY_DAR_012, JHLHY_DAR_013, JHLHY_DAR_015, JHLHY_DAR_074; • Pahang, Cameron Highlands, Brinchang, 18–31.VII.1992, C.W. & L.B. O’Brien, (2, OIST; 8, KUM), JHLHY_DAR_127, JHLHY_DAR_130 – JHLHY_DAR_138.

##### Diagnosis.

Body length 1.7–1.9 mm. Cuticle dark red and largely bare; covered unevenly with yellow scales. Funicle with six articles. Procoxae contiguous. Eyes dimorphic (connected at base of rostrum in male; separated in female). All elytral intervals with erect scales. Erect elytral scales evenly distributed, not concentrated in bundle. Scutellum reduced. Femora without ventral tubercle or tooth. Prosternal cavity very weakly defined and without steep lateral ridges. Metaventrite flattened between meta- and mesocoxae, without a distinct elevated transverse ridge. Metatibial uncus weakly spiral-shaped in male (Fig. 2M–P). Aedeagus wide at midpoint, and tapering near apex (Fig. 15O, P). Internal sac with basal protruding structure (Fig. 15O, P).

##### Distribution.

This species is currently only known from Malaysia (Brinchang).

##### Etymology.

The specific name *nudus* is a Latin adjective that refers to the distinctly naked (unscaled) cuticle of this species.

#### ﻿ Aphanerostethus spinosus

Taxon classificationAnimaliaColeopteraCurculionidae

Lewis & Kojima sp. nov.

FE5E406E-56EA-57BE-8A0B-F9271F6C27DD

https://zoobank.org/7D1FBE1A-4F4B-423B-82D1-57D3ADE821F7

Figs 2I–L , 4F , 15Q, R , 20C, D

##### Specimens examined.

***Holotype*: Malaysia**: • Borneo Island, Sabah, Kinabalu Park Headquarters, alt. 1800–2500 m, 15.III.1993, H. Hiratate, male deposited in KUM, JHLHY_DAR_077.

##### Diagnosis.

Body length 1.9 mm. Cuticle coated in dark, sandy gray, and white scales in indistinct pattern. Funicle with six articles. Procoxae contiguous. Second and odd-numbered elytral intervals with erect scales. Erect elytral scales concentrated in small bundles of two or three along second elytral interval at midpoint; evenly distributed along other intervals. Elytral interval 5 + 6 not distinctly arched at base. Femora all with large, acute tooth along ventral edge. Scutellum distinct, bulging, and covered in white scales. Prosternal cavity prominent and with steep lateral ridges. Metaventrite with a distinct elevated transverse ridge separating the meta- and mesocoxae. Metatibial uncus of male claw-shaped (Fig. 2I–L). Aedeagus with diagnostic, laterally expanded apex (Fig. 15Q, R). Internal sac with M-shaped, basal protruding structure (Fig. 15Q, R).

##### Distribution.

This species is only known from one site in Kinabalu Park, Borneo.

##### Etymology.

This specific name *spinosus* is a Latin adjective in reference to the distinctly acute, elongate tooth on the ventral side of the femora.

##### Comments.

Although the female is unknown it is likely the case that the metatibial uncus is sexually dimorphic as in other closely related *Aphanerostethus* species.

#### ﻿ Aphanerostethus taiwanus

Taxon classificationAnimaliaColeopteraCurculionidae

Lewis, Fujisawa & Kojima sp. nov.

BFCC3373-605C-588F-A77C-0B367FE08D75

https://zoobank.org/61696EC1-39CD-4745-B798-04456E89B9A6

**Figure 21.**
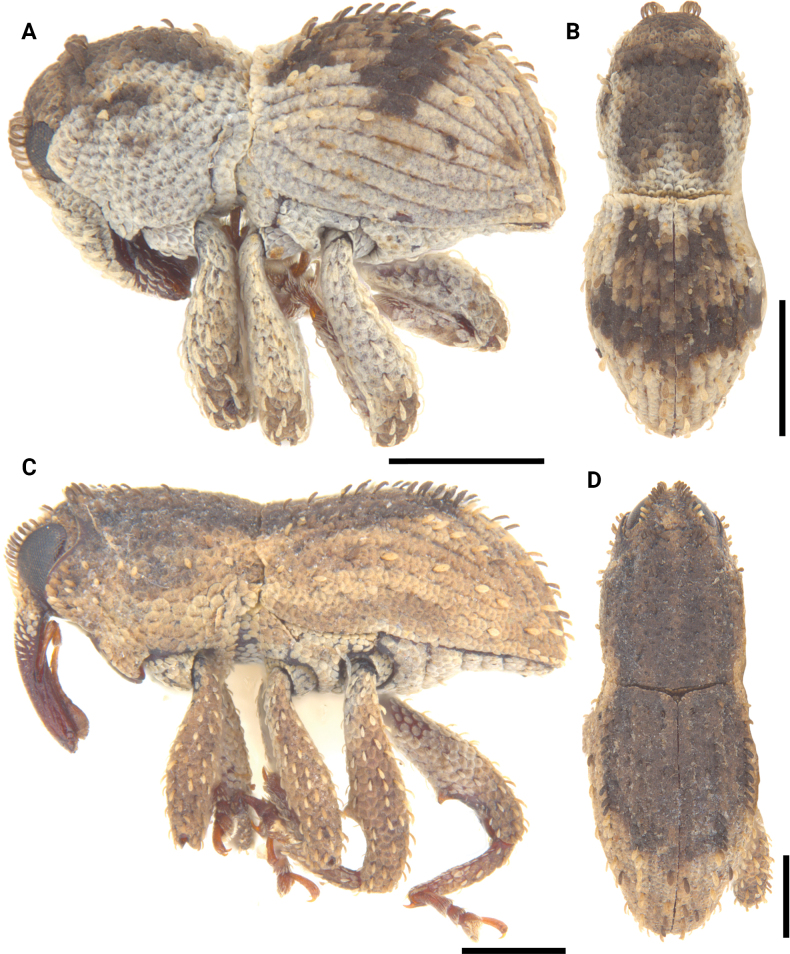
Lateral and dorsal photographs of *Aphanerostethus* species **A, B***Aphanerostethustaiwanus* sp. nov. (JHLHY_DAR_084) **C, D***Aphanerostethusvannideki* Voss, 1957 (JHLHY_DAR_081). Scale bars: 0.5 mm.

Figs 3 , 8D–F , 10F , 15S, T , 21A, B

##### Specimens examined.

***Holotype*: Taiwan**: • Tainan Hsein, Kuanzruling, 6.IV.1965, S. Ueno, male deposited in KUM, JHLHY_DAR_016. ***Paratypes*: Taiwan**: • Taipei Hsein, Kuanzruling, Yangmingshan, 28.III.1965, Y. Hirashima (1, KUM), JHLHY_DAR_017; • Ping Tung Hsein, Kenting, 23.IV.1965, S. Miyamoto, (1, KUM), JHLHY_DAR_018; • Kaohsiung City, Liouguei District, Zhong-Xing-Long Li (near Mt. Taiyuanshan), 19.X.2015, H. Yoshitake, (1, NMNST), JHLHY_DAR_019 (EGP0160E02); • Pingtung Hsein, Kenting, 4.IV.1965, T. Saigusa (2, KUM; 1, OIST), JHLHY_DAR_021, JHLHY_DAR_036, JHLHY_DAR_070; • Pingtung City, Shan Shimen village, Mt. Kao-shih-fo, 14.VII.2014, Y. Komeda, (1, KUM), JHLHY_DAR_129; • Mudan Township, Pingtung County, 22°06'13.46"N, 120°47'33.88"E, 5.III.2016, Y. Fujisawa, (1, TUA), JHLHY_DAR_600; • Mudan Township, Pingtung County, 22°08'21.71"N, 120°51'31.23"E, 9.III.2016, Y. Fujisawa, (1, TUA), JHLHY_DAR_601; • Mudan Township, Pingtung County, 22°05'23"N, 120°47'58"E, 6.III.2016, Y. Fujisawa & S. Shimizu, (2, TUA), JHLHY_DAR_602, JHLHY_DAR_603; • Pingtung Hsein, Kenting, 22–26.II.1982, T. Lin and S.C. Lin, (8, TARI), JHLHY_DAR_083–JHLHY_DAR_090.

##### Diagnosis.

Body length 1.7–1.9 mm. Cuticle coated in dark, sandy gray, and white scales in contrasty pattern. Funicle with six articles. Procoxae contiguous. Second and odd-numbered elytral intervals with sub-erect scales. Erect elytral scales evenly distributed, not concentrated in bundle. Femora without ventral teeth. Scutellum reduced. Prosternal cavity prominent and with steep lateral ridges. Metaventrite with a distinct elevated transverse ridge separating the meta- and mesocoxae. Metatibial uncus simple in both sexes. Aedeagus evenly curved at apex, and in lateral view (Fig. 15S, T). Internal sac without prominent basal protruding structure (Fig. 15S, T).

##### Distribution.

This species is known from a few sites in Taiwan (Kuanzruling, Kenting, Zhong-Xing-Long Li).

##### Etymology.

The specific name is an adjective in reference to the collection locality of the species.

##### Comments.

Our ML analysis (Fig. 3) consistently clustered *A.taiwanus* with *A.distinctus* (BS: 100); however, the presumed basal position of *A.taiwanus* (i.e., sister to *A.distinctus*) was only weakly supported (BS: 55, PP: 0.84). Here, we separate *A.taiwanus* from *A.distinctus* based not on this weak molecular evidence, but primarily on consistent differences in morphology and biogeography. In particular, *A.taiwanus* possesses an oblique row of erect scales on the pronotum (*A.distinctus* with at most 4 or 5 recumbent scales), sub-erect scales on the odd and second elytral intervals (*A.distinctus* with recumbent scales only on odd elytral intervals), differences in male genitalia, and apparently non-overlapping geographic range (*A.taiwanus* only known from Taiwan; *A.distinctus* only known from Japan).

#### ﻿ Aphanerostethus vannideki

Taxon classificationAnimaliaColeopteraCurculionidae

Voss, 1957

1A55E5DE-4AD7-52D4-AA87-A42D0C6B3734

Figs 9A–D , 15U, V , 21C, D

##### Type material examined.

***Neotype* (designated here): Indonesia: West Java**: • Depok, 18.XII.1948, C. Van Nidek, (1, RMNH), ZMAN type COLE.1673.1, JHLHY_DAR_191, bears red label reading “PARATYPUS” as well as new, red neotype label. ***Paratypes*: Indonesia: West Java**: • Depok, 29.II.1948, C. Van Nidek, (1, ZMH), ZMH 841850, bears red label reading “PARATYPUS”; • Depok, 12.IX.1948, C. Van Nidek, (1, ZMH), ZMH 841852, bears original red label reading “PARATYPUS”.

##### Notable historical material examined.

**Indonesia: West Java**: • Depok, 31.VII.1948, C. Van Nidek, (1, ZMH), ZMH 841853, bears red label reading “PARATYPUS”; • Depok, 12.I.1949, C. Van Nidek, (1, ZMH), ZMH 841851, bears red label reading “PARATYPUS”.

##### Non-type material examined.

**Indonesia: West Java**: • Depok, X–XII.1949, C. Van Nidek, (5, RMNH), ZMA.INS.5117697 – ZMA.INS.5117700; **Malaysia**: • Santubon, Kuching, Sarawak, 8.V.1997, T. Takano, (1, KUM), JHLHY_DAR_001; • Sepilok, Sandakan, Sabah, 23.VII–4.VIII.1981, K. Morimoto, (1, KUM), JHLHY_DAR_082; • Sabah, 10.5 miles from Keningau, 6–10.III.1993, H. Kojima, (1, KUM), JHLHY_DAR_081.

##### Diagnosis.

Body length 2.1–2.4 mm. Cuticle covered in dark to pale brown scales, with dark, V-shaped band across anterior part of elytra. Funicle with six articles. Prosternal cavity prominent and with steep lateral ridges. Procoxae separated, and bordered posteriorly by two large projections which receive the rostrum in repose. Femora with prominent ventral teeth. Metaventrite with a distinct elevated transverse ridge separating the meta- and mesocoxae. Metatibial uncus simple in both sexes. Aedeagus tapering at apex, and abruptly curved ventrally at apex in lateral view (Fig. 15U, V).

##### Distribution.

This species is known from Indonesia (West Java) and Malaysia (Sabah, Sarawak).

##### Comments.

Voss (1957) mentions four type specimens in his original description, three of which (ZMH 841852, ZMH 841850, ZMAN type COLE.1673.1) were examined here, as the collection dates / locality / collector data matches the specimens listed in Voss’s description. These three specimens bear red labels reading “PARATYPUS”, suggesting that there is also a holotype; however, no holotype is mentioned in Voss’s paper and we could not locate the holotype. Two additional specimens in the ZMH collection (ZMH 841853, ZMH 841851) with identical locality and collector data (Depok, C. Van Nidek) bear the same red paratype labels, but have the collection dates “31-7-48” and “12-1-49” which do not appear in Voss’s original description. As no holotype was clearly designated, and as we were unable to locate any holotype, we hereby fix the identity of *A.vannideki* by designating one of Voss’s date-verified paratypes (ZMAN type COLE.1673.1, JHLHY_DAR_191) as a neotype for this species.

### ﻿Key to the species of *Aphanerostethus*

*The characters listed in this key are either clearly visible under a light microscope (i.e., external) or genital (requires dissection) in nature, and do not require the use of X-ray microtomography.

**Table:** 

1	Prosternal cavity weak and lacking prominent lateral ridges (Fig. 13A,B); metaventrite at anterior margin of metacoxae without a distinct transverse ridge separating the meta- and mesocoxae (Fig. 18B)	**2**
–	Prosternal cavity prominent and defined by protruding lateral ridges (Fig. 13C, D); metaventrite at anterior margin of metacoxae with a distinct transverse ridge separating the meta- and mesocoxae in most species (Fig. 18A)	**4**
2	Body largely naked (dark red cuticle), except for scattered yellow and brown scales (Fig. 12C, D); erect scales on all elytral intervals; eyes dimorphic (contiguous in males, separated in females); metatibial uncus weakly spiral-shaped in male (Fig. 2M–P)	***A.nudus* sp. nov.**
–	Body covered entirely in pale to dark gray, white, and yellowish scales (Figs 12A, B, 16C, D); erect scales only on odd elytral intervals; eyes separated to same extent in both sexes; metatibial uncus simple in both sexes	**3**
3	Tooth along ventral edge of femora low and obtuse (Fig. 13F; often obscured by scales); scutellum visible; funicle with six articles	***A.japonicus* sp. nov.**
–	Tooth along ventral edge of femora elongate and thorn-like (Fig. 13E); scutellum reduced, barely visible; funicle with five articles	***A.armatus* sp. nov.**
4	Fore coxae separate; posterior edge of prosternum with large projections that receive the rostrum in repose	***A.vannideki* Voss, 1957**
–	Fore coxae contiguous in most species (slightly separated in *A.bifidus*); posterior edge of prosternum lacking projections that receive the rostrum in repose	**5**
5	Elytra with erect, sub-erect or recumbent scales on odd intervals and at least a few on second interval (worn off in some specimens)	**6**
–	Elytra with erect, sub-erect, or recumbent scales on odd intervals only	**9**
6	Scutellum reduced and indistinct; without ventral tooth on hind femur; metatibial uncus simple (unmodified) in males	***A.taiwanus* sp. nov.**
–	Scutellum large and distinct; with prominent ventral tooth on hind femur; metatibial uncus modified in males	**7**
7	Smaller (1.9 mm), gray-scaled, and rounded species; metatibial uncus of male forming a large plate with a distinct apical hook (Fig. 2I–L)	***A.spinosus* sp. nov.**
–	Larger (2.6–3.0 mm), brown-scaled, and elongate species; metatibial uncus of males bifid or ear-shaped (Figs 1A–D, 2A–D)	**8**
8	Metatibial uncus of male ear-shaped (Fig. 2A–D); pronotum approximately 50% the length of elytra; aedeagus (Fig. 15K–L) parallel-sided (in dorsal view), quadrate at apex, and evenly curved (in lateral view)	***A.magnus* sp. nov.**
–	Metatibial uncus of male bifid (Fig. 1A–D); pronotum approximately 60% the length of elytra; aedeagus (Fig. 15A, B) not parallel sided (in dorsal view), tapering at apex, and not evenly curved (in lateral view)	***A.bifidus* sp. nov.**
9	Hind-femur without ventral tooth; standing scales on odd elytral intervals curled back towards body and recumbent; metatibial uncus of male simple (unmodified)	***A.distinctus* (Morimoto & Miyakawa, 1985)**
–	Hind femur with ventral tooth (minute or absent in some *A.morimotoi*); standing scales on odd elytral intervals sub-erect or erect; metatibial uncus modified in known males	**10**
10	Larger (2.2 mm), dark brown scaled species; fore-femur with large, prominent ventral tooth; first elytral interval with prominent, dense cluster of erect scales at midpoint	***A.decoratus* sp. nov.**
–	Smaller (1.7–2.1 mm), grayish-scaled species; fore-femur with or without prominent ventral tooth; first elytral interval with erect scales more or less evenly distributed across elytral length	**11**
11	Third elytral interval with prominent, dense cluster of 8–10 erect scales near midpoint; fore-femur with prominent ventral tooth; fifth elytral interval distinctly arched laterally at elytral base; metatibial uncus of male abruptly curved inwards at midpoint and lacking knob-like projection at apex (Fig. 1M–P)	***A.incurvatus* sp. nov.**
–	Third elytral interval with erect scales more or less evenly distributed across elytral length; fore-femur with or without prominent ventral tooth; fifth elytral interval not arched laterally at elytral base; metatibial uncus of male unknown or not abruptly curved inwards at midpoint and with knob-like projection at apex	**12**
12	Hind-femur with minute ventral tooth or small swelling; metatibial uncus of male truncated at apex and with lateral projection (Fig. 2E–H); standing scales of first and third elytral intervals short, round, sub-erect in anterior half of elytra and elongate and erect in posterior half of elytra; apex of aedeagus elongate and tapering (Fig. 15M, N)	***A.morimotoi* sp. nov.**
–	Hind femur with prominent ventral tooth; standing scales of first and third elytral intervals erect throughout elytral length; apex of aedeagus tapering over apical half, and swelling at tip (Fig. 15E, F) (aedeagus unknown in *A.darlingi*)	**13**
13	Metaventrite at anterior margin of metacoxae with a prominent transverse ridge separating the meta- and mesocoxae; possesses dark and pale, warm brown scales	***A.falcatus* sp. nov.**
–	Metaventrite at anterior margin of metacoxae with a minute tubercle separating the meta- and mesocoxae; with only pale gray and white scales	***A.darlingi* sp. nov.**

### ﻿Concluding remarks

**Figure 22.**
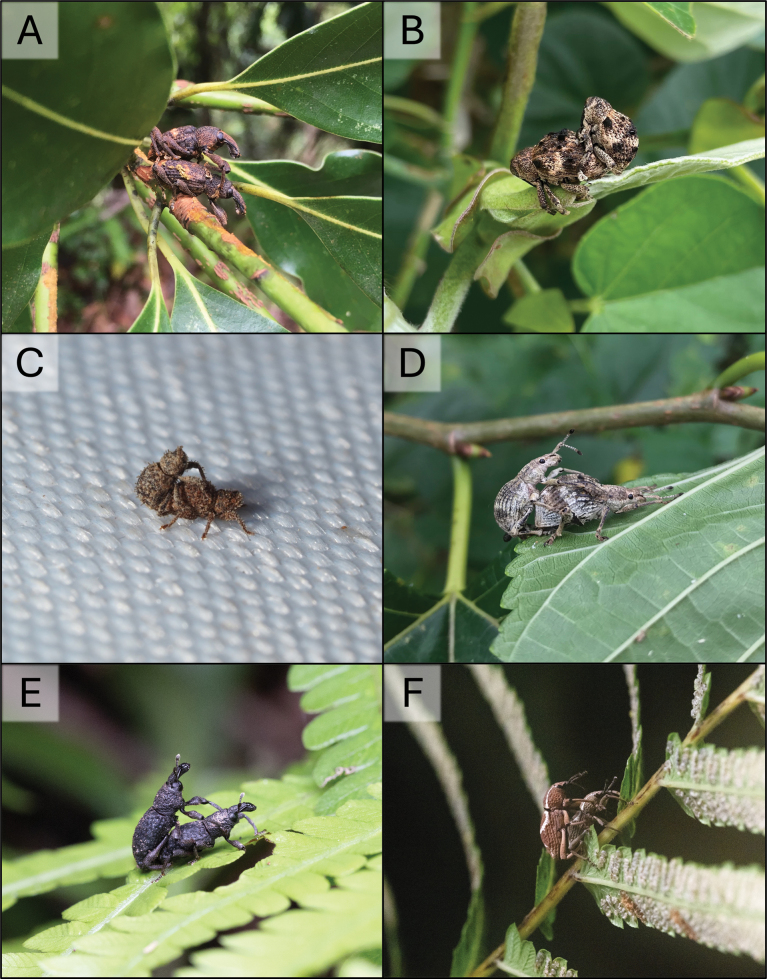
Weevils in copula **A***Pimelocerushylobioides* (Desbrochers, 1891) (Molytinae) (photo credit: JHL) **B***Desmidophoruscrassus* Hubenthal, 1917 (Brachycerinae) (photo credit: JHL) **C***Microcryptorhynchus* sp. (Molytinae) (photo credit: HK) **D***Episomusmori* Kono, 1928 (Entiminae) (photo credit: JHL) **E***Euthycus* sp. (Molytinae) (photo credit: JHL) **F***Cryptodermakuniyoshii* Morimoto, 1978 (Dryophthorinae) (photo credit: JHL). Males often use their hindlegs to grip females from behind, and the metatibiae are usually positioned along the females ventrites or near her genitalia during copulation. The independent evolution of modified, species-specific metatibial unci in males in many weevil lineages may be explained by sexual selection (as they may be used to stimulate females) or as they provide additional gripping function during copulation.

X-ray microtomography was effectively used to examine minute (< 50 μm), frequently obscured metatibial unci in fine detail, and to find stable interspecific differences in cuticle sculpturing and internal (hindwing) morphology in *Aphanerostethus* weevils. Some of these *cryptic* characters are synapomorphies for particular clades (e.g., 10^th^ stria reduction in the *A.distinctus* / *A.taiwanus* clade), and are therefore not only useful for diagnostic purposes, but also for phylogenetics. X-ray μCT adds new dimensions (literally) to the character discovery process, and much like DNA barcoding, will inevitably become standard practice in taxonomy and phylogenetics as the technology becomes more accessible (through outsourcing for most institutions), cheaper, and faster (see van de Kamp et al. 2013). Remarkably, four new *Aphanerostethus* species described here occurred sympatrically on the same Vietnamese mountain (Mt. Lang Biang) and are currently only known from that region. Given that pockets of hitherto undescribed *Aphanerostethus* diversity like this exist, we suspect that the knowledge of *Aphanerostethus* diversity is still in the early stages and that further species will likely be uncovered in other regions. Additionally, seven of the fourteen known *Aphanerostethus* species show species-specific sexual dimorphism in the metatibial uncus (modified in males, simple in females). Many weevils (including Molytinae) possess a simple, straight or curved tooth at the apex of the metatibiae; however, in most groups the shape of this tooth does not vary significantly between sexes or species. Metatibial uncus variation has, however, been documented in several unrelated lineages and exhibit varying degrees of sexual dimorphism, interspecific variation, or both. Documented groups in which such variation occurs includes *Anthonomus* Germar, 1817 (Curculionidae: Curculioninae: Anthonomini) (Eller 1995), *Conotrachelus* Dejean, 1835 (Curculionidae: Molytinae: Conotrachelini) (Schoof 1942), *Lignyodes* Dejean, 1835 (Curculionidae: Curculioninae: Tychiini) (Clark 1980a, b; Clark and Lodos 1981), *Lissorhoptrus* (Curculionidae: Brachycerinae: Tanysphyrini) (Kuschel 1951; O’Brien and Haseeb 2014), *Plocetes* LeConte, 1876 (Curculionidae: Curculioninae: Tychiini) (Clark 1980c, 1982), *Proctorus* LeConte, 1876 (Curculionidae: Curculioninae: Ellescini) (Lewis and Anderson 2022), and *Tyloderma* Say, 1832 (Curculionidae: Molytinae: Cryptorhynchini) (Wibmer 1981). Although the independent evolution of species-specific metatibial unci in multiple weevil lineages has not been formally investigated, we suspect that, as the metatibial unci would be positioned near the rear (i.e., genitalia) of the female during copulation (Fig. 22), the unci may be used to stimulate the female (sexual selection). This selection mechanism would explain why metatibial uncus variation is apparently more common than pro- and mesotibial unci variation. Alternatively, they may provide additional gripping function during copulation (see Haley and Gray (2011) (coercion) and Harari et al. (2003) (mate guarding)). Careful observation of weevil mating behavior comparing lineages with and without modified metatibial unci would be lucrative and help confirm the above hypothesis.

## Supplementary Material

XML Treatment for Aphanerostethus

XML Treatment for Aphanerostethus armatus

XML Treatment for Aphanerostethus bifidus

XML Treatment for Aphanerostethus darlingi

XML Treatment for Aphanerostethus decoratus

XML Treatment for Aphanerostethus distinctus

XML Treatment for Aphanerostethus falcatus

XML Treatment for Aphanerostethus incurvatus

XML Treatment for Aphanerostethus japonicus

XML Treatment for Aphanerostethus magnus

XML Treatment for Aphanerostethus morimotoi

XML Treatment for Aphanerostethus nudus

XML Treatment for Aphanerostethus spinosus

XML Treatment for Aphanerostethus taiwanus

XML Treatment for Aphanerostethus vannideki

## ﻿Additional information

### Author ORCIDs

Jake H. Lewis https://orcid.org/0000-0001-5147-7237

Dimitrios Petsopoulos https://orcid.org/0000-0002-4408-0458

Yusuke Fujisawa https://orcid.org/0009-0007-1758-7599

Xuan Lam Truong https://orcid.org/0000-0002-1758-903X

Dan L. Warren htt https://orcid.org/0000-0002-8747-2451 ps

### Data availability

All of the data that support the findings of this study are available in the main text or Supplementary Information.

## Supplementary materials

Supplementary material 1 Primer and thermocycler settings for *Aphanerostethus* barcoding Data type xlsx https://binary.pensoft.net/file/1163087This dataset is made available under the Open Database License (http://opendatacommons.org/licenses/odbl/1.0/). The Open Database License (ODbL) is a license agreement intended to allow users to freely share, modify, and use this Dataset while maintaining this same freedom for others, provided that the original source and author(s) are credited.Author: Miyuki Suenaga
